# Supplementary material for: Gas-responsive porous magnet distinguishes the electron spin of molecular oxygen
Source: Nat Commun. 2018 Dec 21;9:5420. doi: 10.1038/s41467-018-07889-1 (PMC6303325; doi:10.1038/s41467-018-07889-1)
Supplement: Supplementary file 1 — Supplementary Information [file 41467_2018_7889_MOESM1_ESM.pdf]

Supplementary Information

Gas-responsive porous magnet distinguishes the electron spin of molecular oxygen

Wataru Kosaka[1,2], Zhaoyuan Liu[2], Jun Zhang[2], Yohei Sato[3], Akihiro Hori[4], Ryotaro Matsuda[4], Susumu Kitagawa[3] & Hitoshi Miyasaka*[1,2]

[1] Institute for Materials Research, Tohoku University, 2-1-1 Katahira, Aoba-ku, Sendai 980-8577, Japan. E-mail: miyasaka@imr.tohoku.ac.jp

[2] Department of Chemistry, Graduate School of Science, Tohoku University, 6-3 Aramaki-Aza-Aoba, Aoba-ku, Sendai 980-8578, Japan

[3] Institute for Integrated Cell-Materials Science (iCeMS), Kyoto University, Katsura, Nishikyo-ku, Kyoto 615-8510, Japan

[4] Department of Materials Chemistry, Graduate School of Engineering, Nagoya University, Furo-cho, Chikusa-ku, Nagoya 464-8603, Japan

*Corresponding author
Hitoshi Miyasaka
Institute for Materials Research, Tohoku University
2-1-1 Katahira, Aoba-ku, Sendai 980-8577, Japan
Tel: +81-22-215-2030
Fax: +81-22-215-2031

E-mail: miyasaka@imr.tohoku.ac.jp

Supplementary Methods

Materials. All chemicals were purchased from commercial sources and were of reagent-grade quality. Dichloromethane (DCM) and 1,2-dichloroethane (DCE) were distilled under a N_2 atmosphere using common drying agents. [Ru_2(3,5-$F_2PhCO_2)_4(THF)_2$] was synthesized by literature methods.[1]

Synthesis of [{Ru_2(3,5-$F_2PhCO_2)_4$}$_2${TCNQ(MeO)$_2$}]·3(DCM)·1.5(DCE) (1-solv) and its solvent-free compound (1). A DCM solution (40 mL) of [Ru_2(3,5-$F_2PhCO_2)_4(THF)_2$] (146 mg, 0.15 mmol) was separated into 2 mL portions and placed in narrow-diameter glass tubes (inner diameter of 8 mm) as the bottom layer. Then, a mixed solvent of DCM/DCE 1:1 v/v (0.5 mL) was added on the bottom layer to slow the rate of diffusion (middle layer). Finally, a DCE solution (40 mL) of TCNQ(MeO)$_2$ (19.8 mg, 0.075 mmol) was separated into 2 mL portions and placed onto each middle layer (top layer) and sealed for diffusion to occur. The glass tubes were left undisturbed for one week to obtain plate-shaped black crystals of **1-solv** (yield 9%). X-ray crystallography and thermogravimetric measurements indicated that the crystals contained crystallization solvents (i.e., 3 DCM and 1.5 DCE) that were easily eliminated after exposure to air, making the elemental analysis data difficult to interpret. Therefore, elemental analysis was performed only for the solvent-free dried sample **1**. IR (KBr): ν(C≡N), 2187, 2166 cm^{-1}. Crystal samples of **1** were prepared by heating **1-solv** at 353 K under vacuum for 12 h. Elemental analysis (%) calcd for $C_{70}H_{32}F_{16}N_4O_{18}Ru_4$: C 43.67, H 1.69, N 2.91. Found: C 43.13, H 1.74, N 2.99. IR (KBr): ν(C≡N), 2197, 2127 cm^{-1}. Unless otherwise stated, the pristine sample of **1** discussed as a porous magnet was prepared by heating at 353 K under vacuum for 12 h.

Single-crystal X-Ray crystallographic analysis for 1-solv and 1. Crystal data for **1-solv** and **1** were collected at 134 K and 112 K, respectively, on a CCD diffractometer (Rigaku Saturn724) with multi-layer mirror monochromated Mo-$K\alpha$ radiation (λ = 0.71075 Å). A single crystal was mounted on a thin Kapton film using Nujol and cooled in a N_2 gas stream. The structures were solved using direct methods (SIR2004), which were expanded using Fourier techniques and refined by full-matrix least-squares refinements on F^2. The non-hydrogen atoms were refined anisotropically and hydrogen atoms were refined using a riding model. These data have been deposited as CIFs at the Cambridge Data Centre as supplementary publication nos. CCDC-1519242 and 1519241 for **1-solv** and **1**, respectively. Copies of the data can be obtained free of charge from the CCDC via www.ccdc.cam.ac.uk/data_request/cif. Structural diagrams were prepared using VESTA software.[2] The void volumes in the crystal structures were estimated using PLATON.[3] To make an easier comparison of the cell constants between **1-solv** and **1**, the unit cell of **1** was transformed following vector alternations as $\boldsymbol{a}' = \boldsymbol{a}$, $\boldsymbol{b}' = -\boldsymbol{c}$, and $\boldsymbol{c}' = -\boldsymbol{a} + \boldsymbol{b}$, where $\boldsymbol{a}$, $\boldsymbol{b}$, and $\boldsymbol{c}$ are the standard axis vectors based on the IUCR rule, and $\boldsymbol{a}'$, $\boldsymbol{b}'$, and $\boldsymbol{c}'$ are the transformed axis vectors with cell constants of a' = 10.8393(11) Å, b' = 14.9486(11) Å, c' = 13.8565(16) Å, α' = 89.887(7)°, β' = 119.034(12)°, and γ' = 92.726(6)° for **1** (depicted as red arrows in Fig. 1c and 1d).

Single-crystal X-Ray crystallographic analysis for 1⊃N_2, 1⊃O_2-I, and 1⊃CO_2. Eventually, crystal data for **1⊃N_2**, **1⊃O_2-I**, and **1⊃CO_2** were collected at 130 K, 130 K, and 195 K, respectively, on a CCD diffractometer (Rigaku Saturn724) with multi-layer mirror monochromated Mo-$K\alpha$ radiation (λ = 0.71073 Å). A single crystal of **1-solv** was mounted on a thin glass capillary with a minimum amount of epoxy adhesive, which was attached to the inner-wall of silica glass capillary with an inner diameter of 0.5 mm. The capillary was connected to gas-handling system through o-ring seal connector based on Swagelok Ultra-Torr® adaptor, which can be fixed on a diffraction goniometer. Then the crystal was evacuated at room temperature

for one hour, 100 kPa of N_2, 10 kPa of O_2, and 100 kPa of CO_2 was introduced at room temperature, then slowly cooled to an appropriate target temperature by a N_2 gas stream. The structures were solved using direct methods (SHELXLT), which were expanded using Fourier techniques and refined by full-matrix least-squares refinements on F^2. The non-hydrogen atoms were refined anisotropically and hydrogen atoms were refined using a riding model. These data have been deposited as CIFs at the Cambridge Data Centre as supplementary publication nos. CCDC-1519243, 1519244, and 1519240 for **1⊃N₂**, **1⊃O₂-I**, and **1⊃CO₂**, respectively. Copies of the data can be obtained free of charge from the CCDC via www.ccdc.cam.ac.uk/data_request/cif. To make an easier comparison of the cell constants between **1-solv** and **1⊃Gas**, the unit cell of **1⊃Gas** was transformed following vector alternations as $\boldsymbol{a}' = -\boldsymbol{a}$, $\boldsymbol{b}' = \boldsymbol{c}$, and $\boldsymbol{c}' = \boldsymbol{a} + \boldsymbol{b}$, where $\boldsymbol{a}$, $\boldsymbol{b}$, and $\boldsymbol{c}$ are the standard axis vectors based on the IUCR rule, and $\boldsymbol{a}'$, $\boldsymbol{b}'$, and $\boldsymbol{c}'$ are the transformed axis vectors with cell constants of a' = 10.5642(18) Å, b' = 15.718(3) Å, c' = 14.078(3) Å, α' = 93.357(17)°, β' = 123.95(3)°, and γ' = 87.664(14)° for **1⊃N₂**, a' = 10.6878(15) Å, b' = 15.895(2) Å, c' = 14.177(2) Å, α' = 94.256(14)°, β' = 123.65(2)°, and γ' = 86.319(12)° for **1⊃O₂-I**, and a' = 10.8173(11) Å, b' = 15.9803(15) Å, c' = 14.2610(17) Å, α' = 95.640(9)°, β' = 122.470(15)°, and γ' = 83.517(8)° for **1⊃CO₂**, (depicted as red arrows in Fig. 2c and Supplementary Fig. 9 and 10).

Structural determination of 1⊃O₂-II from powder X-ray diffraction. A ground sample of **1** was sealed in a silica glass capillary with an inner diameter of 0.5 mm. The powder X-ray diffraction (PXRD) pattern was obtained with a 0.02° step using an Ultima IV diffractometer with Cu-$K\alpha$ radiation (λ = 1.5418 Å) with θ scan. To obtain the PXRD patterns under the gas-adsorbed conditions, the glass capillary was connected to stainless-steel (SUS) lines with valves to dose and remove the gas, which were connected to a gas-handling system (BELSORP max; BEL inc). The temperature was controlled by a N_2 gas stream.

Cell parameters were determined using DIFFRACplus TOPAS® v4.2 software. A Le Bail structureless profile fitting algorithm, which affords refined cell parameters with triclinic space group; $P-1$ space group was postulated.

Ab initio structure solution from diffraction data using a direct-space method was performed using FOX software.[4] On the direct-space method, the orientations of two [Ru_2] units and TCNQ(MeO)$_2$ were varied. Bond lengths and angles of each molecules were fixed except for the dihedral angle between benzene ring and carboxylate bridge of benzoate moiety. Since [Ru_2] unit and TCNQ(MeO)$_2$ have an inversion center, the center of each units were fixed around each inversion center of unit cell. The unit cell of $P-1$ space group has eight crystallographically-unique inversion centers. Only when an appropriate combination of those were assigned to each unit, direct-space method yielded probable structures; among the resultant structures after hundreds of trials, 20~50% of the structures were satisfactory from the viewpoint of the connectivity of [Ru_2] unit and TCNQ(MeO)$_2$; layered structure was formed, which were almost identical each other.

Structural refinement was performed using the Rietveld method with RIETAN-FP software.[5] Refined parameters were as follows; a) Three peak-shift parameters, b) twelve background parameters, c) scale factor, d) peak profile parameters with Split-Pearson VII function (three peak width parameters, three asymmetry parameters, and four decay parameters), e) lattice constants (a, b, c, α, β, γ), f) overall isotropic atomic displacement parameter, and g) fractional coordinates (x, y, z) for non-hydrogen atoms (~60 crystallographically unique non-hydrogen atoms). All the above parameters are refined under the soft constraints on bond distances, bond angles, and dihedral angles throughout the refinement. The parameters were refined incrementally with conjugate-gradient method. On the initial stage of the refinement, hydrogen atoms were removed from the structural model. After all parameters were refined, hydrogen

atoms were attached to the calculated positions, and then, all parameters were refined. Fractional coordinates of hydrogen atoms were not refined. After the refinement, fractional coordinates of hydrogen atoms were again calculated and modified. This process was repeated until the fractional coordinates of hydrogen atoms became self-consistent. On the final refinement, all parameters except for lattice constants and scale factor were fixed to evaluate standard error of the lattice constants. The refinement cycle was estimated from agreement factors of $R_{wp} = [\Sigma w[y-f(\boldsymbol{x})]^2/\Sigma wy^2]^{1/2}$, where y and $f(\boldsymbol{x})$ represent the observed intensity and the calculated intensity at a diffraction angle of 2θ, respectively, and $R_B = \Sigma||F_o| - |F_c||/\Sigma|F_o|$.

These data have been deposited as CIFs at the Cambridge Data Centre as supplementary publication nos. CCDC-1519245 for **1⊃O$_2$-II**, respectively. Copies of the data can be obtained free of charge from the CCDC via www.ccdc.cam.ac.uk/data_request/cif. For easier comparison of the cell constants among the observed phases, the unit cells of **1⊃O$_2$-II** was transformed following vector alternations as $\boldsymbol{a}' = -\boldsymbol{a}$, $\boldsymbol{b}' = \boldsymbol{c}$, and $\boldsymbol{c}' = \boldsymbol{a} + \boldsymbol{b}$, where $\boldsymbol{a}$, $\boldsymbol{b}$, and $\boldsymbol{c}$ are the standard axis vectors based on the IUCR rule, and $\boldsymbol{a}'$, $\boldsymbol{b}'$, and $\boldsymbol{c}'$ are the transformed axis vectors with cell constants of a' = 10.697(2) Å, b' = 15.826(2) Å, c' = 14.633(3) Å, α' = 96.075(19)°, β' = 121.14(3)°, and γ' = 81.95(2)° (depicted as red arrows in Fig. 2c).

Determination of structural phase diagram for 1⊃O$_2$. Compound **1** on a copper plate was placed in a closed-He-cycle refrigerator-based cryostat (400 to 40 K) connected to a gas-handling apparatus (BELSORP-18PLUS; Bel Japan). The cryostat is equipped with beryllium windows, through which PXRD patterns were collected using an Ultima IV diffractometer with Cu-$K\alpha$ radiation (λ = 1.5418 Å) with θ–2θ scan.

***In situ* infrared spectra measurements.** *In situ* infrared (IR) spectral measurements were conducted through diamond and CaF_2 windows on a homemade cryostat system based on the Oxford cryostat MicrostatN and connected to a gas handling and pressure monitoring system with transmission configuration using a JASCO FT-IR 4200 spectrometer. Neat samples were sandwiched between two CaF_2 plates, and heated under vacuum at 350 K for 2 h prior to the measurements.

***In situ* gas adsorption-magnetic measurements.** A schematic illustration of the cell for the *in situ* magnetization measurements under gas is depicted in Supplementary Fig. 12. A known weight (ca. 25 mg) of the dried sample was placed in the bottom of a close-ended quartz tube (inner diameter of 4 mm). A piece of cotton was placed above the sample to prevent sample movement during the gas sorption process. Then, the tube was attached to the edge of a homemade sample rod made of SUS and a brass male thread with fluorocarbon tape. The sample was isolated from the surrounding atmosphere by overlaying a close-ended brass tube, which could be attached to the thread on the sample rod by screwing its open end. An airtight seal between the thread and the brass tube was achieved by a silicon sealant (CAF® 4, Bluestar Silicones). The SUS tube was connected to the gas handling system with a turbo molecular pump and manometer. Magnetic measurements were performed in the same manner as normal measurements using a Quantum Design SQUID magnetometer MPMS; it was needless to subtract the background signal from the brass tube. The sample was evacuated down to 0.1 Pa with a turbomolecular pump at 353 K and the gas was introduced at 200 K up to an inner gas pressure of ~116 kPa. The gas-sealed cell was then cooled with a sweep rate of 0.5 K min^{-1} to 120 K for N_2, 195 K for CO_2, and 100 K for O_2, and then maintained at these respective temperatures for 10 h to reach adsorption equilibrium. Then, the temperature was set to 120 K and the field-cooled magnetization (FCM) measurement was started. In the case of O_2, the effect of increasing the temperature from 100 to 120 K on the magnetic measurement was negligible because desorption is slow in this temperature region, as confirmed by measurement of the M–T curves

between 100–1.8 K (Supplementary Fig. 20), which provided completely identical curves below 100 K to those measured between 120–1.8 K. For the pressure-dependent measurement of the FCM curves under O_2 (Fig. 4a), the pressure was reduced to the designated pressure at 100 K after adsorption equilibrium under 100 kPa of O_2 was achieved at 100 K by the method mentioned above. Then, the FCM measurements were started from 100 K. This process was employed because it takes a considerably long time to achieve adsorption equilibrium at around 100 K in the low O_2 pressure region (< 1 kPa) when O_2 was introduced in **1**. Before demonstration of magnetic switching at 85 K (Fig. 6), the same procedure was applied; after reaching adsorption equilibrium at 100 K under 100 kPa of O_2, the O_2 pressure was reduced to 50 kPa and the temperature was then decreased to 85 K. Despite placing cotton above the sample, a small step was inevitably observed at zero magnetic field in the magnetic hysteresis loop measurement due to slight sample movement, since a fixation reagent (e.g., Nujol oil) could not be used because gas adsorption at cryogenic temperatures was necessary.

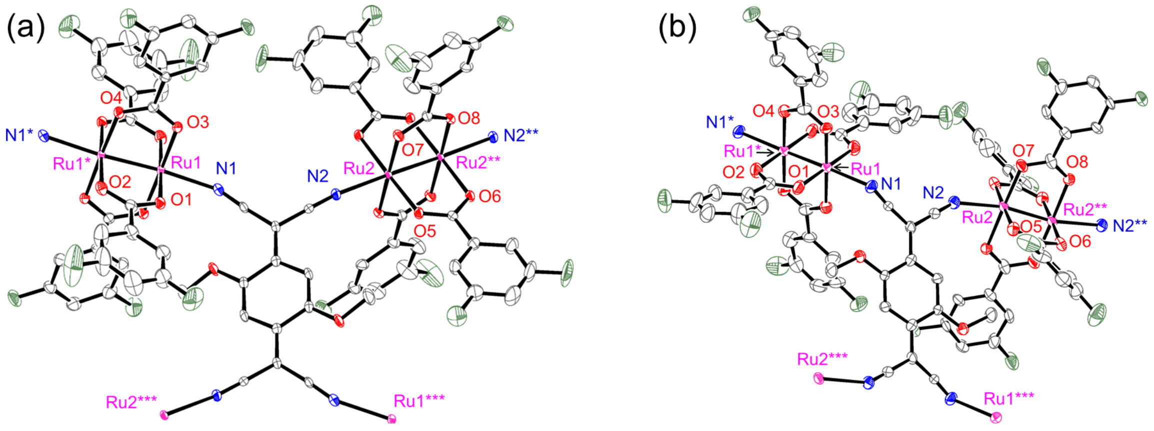

Supplementary Figure 1. Thermal ellipsoid plot obtained from single crystal X-ray diffraction (SCXRD) analyses showing the asymmetric unit and atom numbering scheme for **1-solv** (a) and **1** (b), where O, C, N, F, and Ru are represented in red, gray, blue, green, and purple, respectively. Displacement ellipsoids are drawn at a 50% probability level. Hydrogen atoms and guest molecules are omitted for clarity. Symmetry codes: (*) $-x + 1, -y + 2, -z + 1$ for **1-solv**, $-x + 1, -y + 1, -z$ for **1**, (**) $-x + 1, -y + 1, -z$ for **1-solv**, $-x + 2, -y, -z + 1$ for **1**, (***) $-x + 1, -y + 1, -z + 1$ for **1-solv** and **1**.

Supplementary Table 1. Crystallographic data obtained from SCXRD analyses for **1-solv** and **1**.

	**1-solv**	**1**
Formula	$C_{76.5}H_{41}Cl_{13}F_{16}N_4O_{18}Ru_4$	$C_{70}H_{32}F_{16}N_4O_{18}Ru_4$
formula weight	2473.32	1925.29
crystal system	Triclinic	Triclinic
space group	$P{-}1$	$P{-}1$
a / Å	10.652(2)	10.8393(11)
b / Å	14.666(3)	12.7948(14)
c / Å	15.526(3)	14.9486(19)
α / deg	91.681(2)	87.813(5)
β / deg	108.867(4)	87.274(6)
γ / deg	102.6707(18)	71.242(4)
V / Å^3	2226.0(8)	1960.3(4)
Z	1	1
crystal size / mm^3	0.20×0.15×0.04	0.23×0.12×0.05
T / K	134(1)	112(1)
D_{calc} / g·cm^{-3}	1.845	1.631
F_{000}	1213.00	944.00
λ / Å	0.71075	0.71075
μ(Mo Kα) / cm^{-1}	11.571	8.609
data measured	15138	12584
data unique	7672	6691
R_{int}	0.0189	0.0473
no. of observations	7672	6691
no. of variables	649	505
$R1$ ($I > 2.00\sigma(I)$)^a	0.0408	0.0942
R (all reflections)^a	0.0452	0.1200
$wR2$ (all reflections)^b	0.1264	0.2646
GOF	1.073	1.083
CCDC No.	1519242	1519241

^{a} $R1 = R = \Sigma||F_o| - |F_c||/\Sigma|F_o|$. ^{b} $wR2 = [\Sigma w(F_o^2 - F_c^2)^2/\Sigma w(F_o^2)^2]^{1/2}$

Supplementary Table 2. Selected bond length (Å) and angles (˚) obtained from SCXRD analyses in **1-solv** and **1**, where θ represents dihedral angle between the least squares planes defined by the phenyl ring of benzoate ligand and a carboxylate-bridging mode (atom set of M_2O_2C).

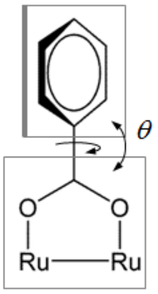

	**1-solv**	**1**
Ru1–O1	2.020(3)	2.043(7)
Ru1–O2a	2.028(3)	2.058(7)
Ru1–O3	2.027(3)	2.035(7)
Ru1–O4a	2.025(3)	2.057(7)
Ru2–O5	2.065(3)	2.097(8)
Ru2–O6b	2.074(3)	2.092(8)
Ru2–O7	2.063(3)	2.099(7)
Ru2–O8b	2.060(3)	2.104(7)
Ru1–N1	2.225(4)	2.263(8)
Ru2–N2	2.292(4)	2.350(8)
Ru1–Ru1a	2.2894(6)	2.3245(12)
Ru2–Ru2b	2.2889(6)	2.3148(10)
Ru1–N1−C29	154.7(3)	132.2(9)
Ru2–N2−C31	165.3(3)	148.4(7)
θ		
benzoate set-1	5.46	10.26
benzoate set-2	14.78	10.80
benzoate set-3	6.03	27.53
benzoate set-4	4.77	6.92

Symmetry codes: (a) $-x + 1, -y + 2, -z + 1$ for **1-solv**, $-x + 1, -y + 1, -z$ for **1**, (b) $-x + 1, -y + 1, -z$ for **1-solv**, $-x + 2, -y, -z + 1$ for **1**.
Benzoate set-1 to -4: the phenyl group of C2−C8, C10−C16, C18−C24 and C26−C32, respectively.

Supplementary Note 1: On the oxidation state of the [Ru_2] unit in 1-solv and 1.

The oxidation state of [Ru_2] unit can be known from the Ru–O_{eq} length (O_{eq} = equatorial oxygen atoms), which is quite sensitive to the oxidation state of the [Ru_2] unit and to be 2.06–2.07 Å for [$Ru_2^{II,II}$] and 2.02–2.03 Å for [$Ru_2^{II,III}$]$^+$.[6] The average Ru–O_{eq} length of **1-solv** is 2.025 and 2.066 Å for [$Ru(1)_2$] and [$Ru(2)_2$] unit, respectively. The average Ru–O_{eq} length of **1** is 2.048 and 2.098 Å for [$Ru(1)_2$] and [$Ru(2)_2$] unit, respectively.

Supplementary Table 3. Comparison of bond lengths (Å) obtained from SCXRD analyses in TCNQ moieties for **1-solv** and **1**.

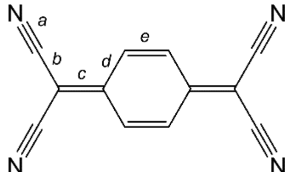

	charge	*a*	*b*	*c*	*d*	*e*	ρ^b	Ref
**I**	0	1.140(1)	1.441(1)	1.374(3)	1.448(4)	1.346(3)	0	7
**II**	−1	1.153(7)	1.416(8)	1.420(1)	1.423(3)	1.373(1)	−1	8
**1-solv**		1.154(6)	1.409(6)	1.431(6)	1.427(7)	1.368(6)	−1.21 [c]	
		1.144(6)	1.418(7)		1.414(7)			
		1.149 [a]	1.414 [a]		1.420 [a]			
**1**		1.190(12)	1.411(13)	1.451(13)	1.444(12)	1.386(12)	−1.26 [c]	
		1.164(11)	1.438(12)		1.440(13)			
		1.177 [a]	1.424 [a]		1.442 [a]			

I: TCNQ, **II**: RbTCNQ. [a]average value, [b]estimated from the average values, [c]$\rho = A_H[c/(b + d)] + B_H$ with A_H = −41.667 and B_H = 19.833.

Supplementary Note 2: Additional description on the crystal structure of 1-solv and 1.

In **1-solv**, the D_2A layers interact each other through π stacks between the phenyl rings on the benzoate moieties of [Ru_2] units (distances between π-planes on [$Ru(1)_2$] and [$Ru(2)_2$] units were 3.28 and 3.17 Å, respectively). Whereas, such π stacks was disappeared in **1**.

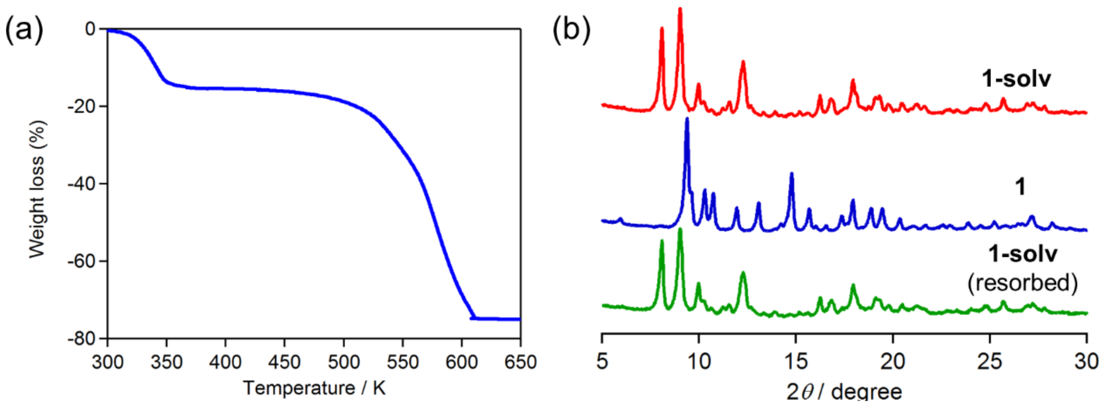

Supplementary Figure 2. (a) Thermogravimetric analyses profiles of **1-solv** with a heating rate of 5 K min^{-1}. (b) PXRD patterns of **1-solv** (red), **1** obtained by drying **1** in vacuo at 353 K for 12 h (blue), and **1** exposed to DCE vapor at 298 K for 48 h (green, i.e., **1-solv** (resorbed)), Cu-*K*α radiation with λ = 1.54 Å.

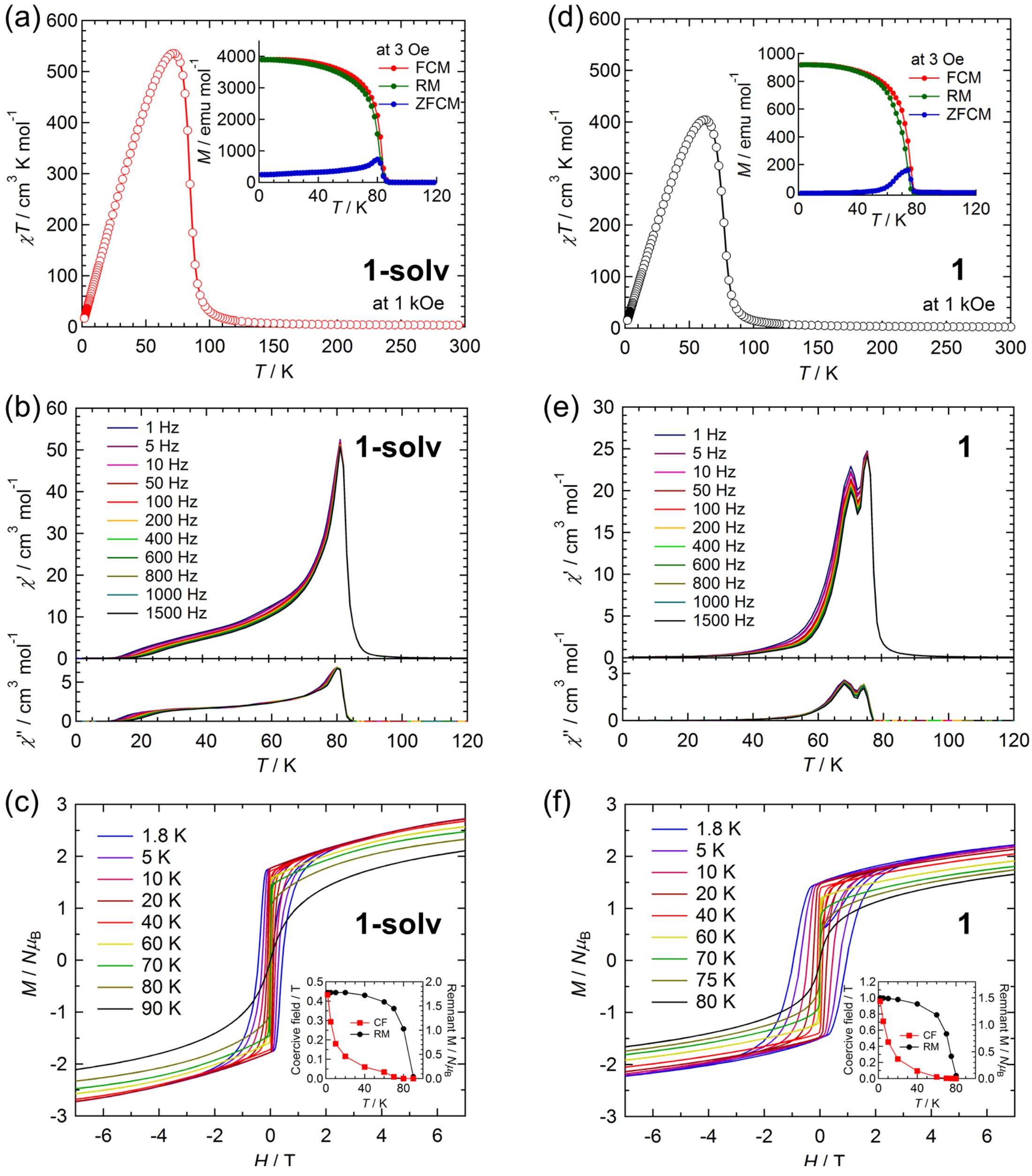

Supplementary Figure 3. Temperature dependence of χT for **1-solv** (a) and **1** (d) measured under dc field of 1 kOe. Inset: *M*–*T* curves measured under dc fields of 3 Oe. Temperature dependence of ac magnetic susceptibilities χ' (in-phase) and χ'' (out-of-phase) at zero dc field and 3 Oe ac oscillating field for **1-solv** (b) and **1** (e). Field dependence of magnetization of **1-solv** (c) and **1** (f) at several temperatures between 1.8 K and 90 K. Inset: temperature dependence of coercive field and remnant magnetization.

Supplementary Note 3: Ac magnetic measurements for 1-solv and 1.

To obtain more details of the spin ordering, temperature dependence of the ac magnetic susceptibility (χ', real part, χ'', imaginary part) was measured under a zero dc field and a 3 Oe oscillating field in the frequency range from 1 to 1.5 kHz (Supplementary Fig. 3b for **1-solv**, Supplementary Fig. 3e for **1**). The χ' value for **1-solv** exhibits a single distinct peak at 81 K without any noticeable frequency dependence at the maximum accompanied by an increase in χ'' at 83 K, indicating the onset of long-range ferrimagnetic ordering with T_C = 83 K. The χ' value for **1** exhibits a single distinct peak at 75 K without any noticeable frequency dependence at the maximum accompanied by an increase in χ'' at 76 K, indicating the onset of long-range ferrimagnetic ordering with T_C = 76 K. The other peak observed in both χ' and χ'' at 70 K may indicate the involvement of phase transition to a new spin-canted state.

Supplementary Note 4: About *M–H* for 1-solv and 1.

Supplementary Fig. 3c and 3f shows the field-dependence of the magnetization (*M–H*) for **1-solv** and **1**, respectively, measured at various temperatures in the field range of ±7 T. The saturated magnetization (M_s) value at 7 T for the 1.8 K data of **1-solv** and **1** is 2.73 and 2.22 $N\mu_B$, respectively. The slightly smaller M_s value in **1** compared to that in **1-solv** implies ordered spins somehow make a canting form, which could be due to the structural change upon the desolvation, probably associated with the deformation of arrangement of magnetic anisotropic planes for [Ru_2] units, as realized from the dihedral angle of the planes (Supplementary Table 8). Relatively large hysteresis loops with coercive field of 0.43 and 0.96 T for **1-solv** and **1**, respectively, at 1.8 K concludes that these compounds have large magnetic anisotropy arisen from composed [Ru_2]$^{0/+}$ units and their layered features. The coercive field for **1-solv** and **1** quasi-exponentially decreases with increasing temperature and finally disappears at around 80 K, corresponding to T_C (inset of Supplementary Fig. 3c and 3f for **1-solv** and **1**, respectively). The remnant magnetization (RM) begins to decrease at temperatures above 70 and 60 K for **1-solv** and **1**, respectively (inset of Supplementary Fig. 3c and 3f for **1-solv** and **1**, respectively).

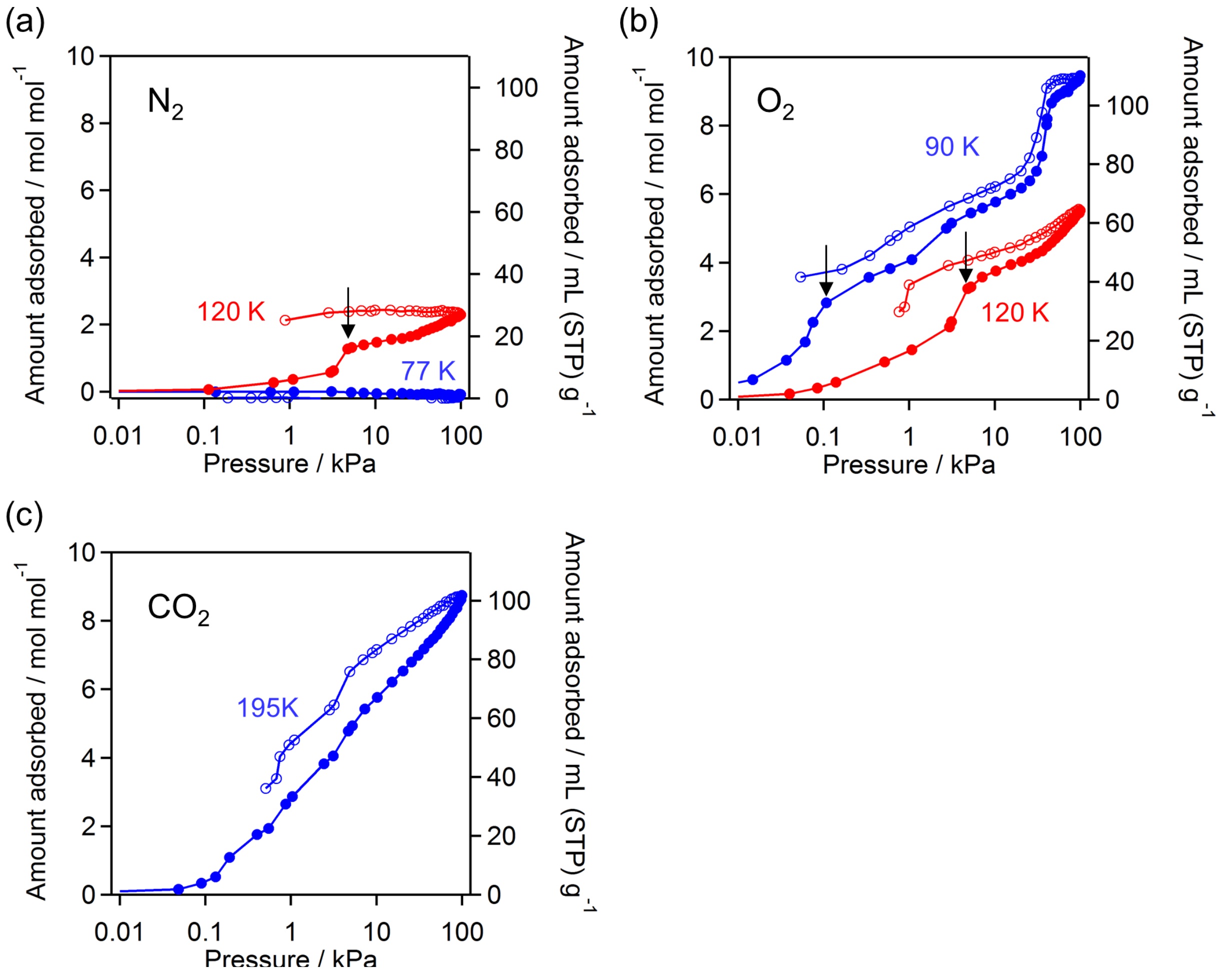

Supplementary Figure 4. Adsorption (closed) and desorption (open) isotherms of **1** in a log-scale for N_2 (a), O_2 (b) and CO_2 (c). Black arrows indicate the occurrence of 1st gate-opening adsorption.

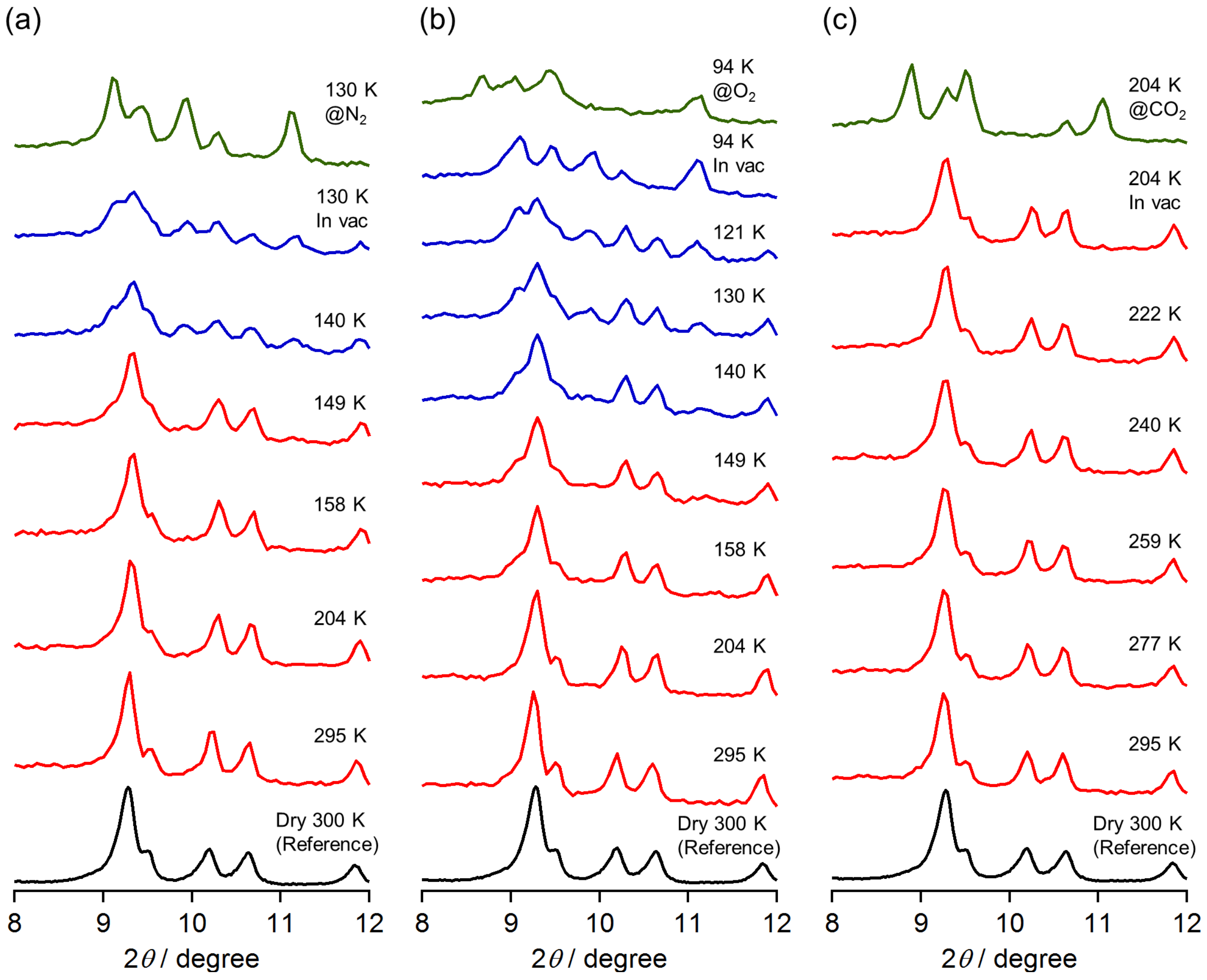

Supplementary Figure 5. Temperature dependences of PXRD pattern (λ = 1.54 Å) measured on heating in vacuum after N_2 adsorption at 130 K (a), O_2 adsorption at 94 K (b) and CO_2 adsorption at 204 K. These results confirmed the structural reversibility of **1** upon sorption-desorption cycles for these gases.

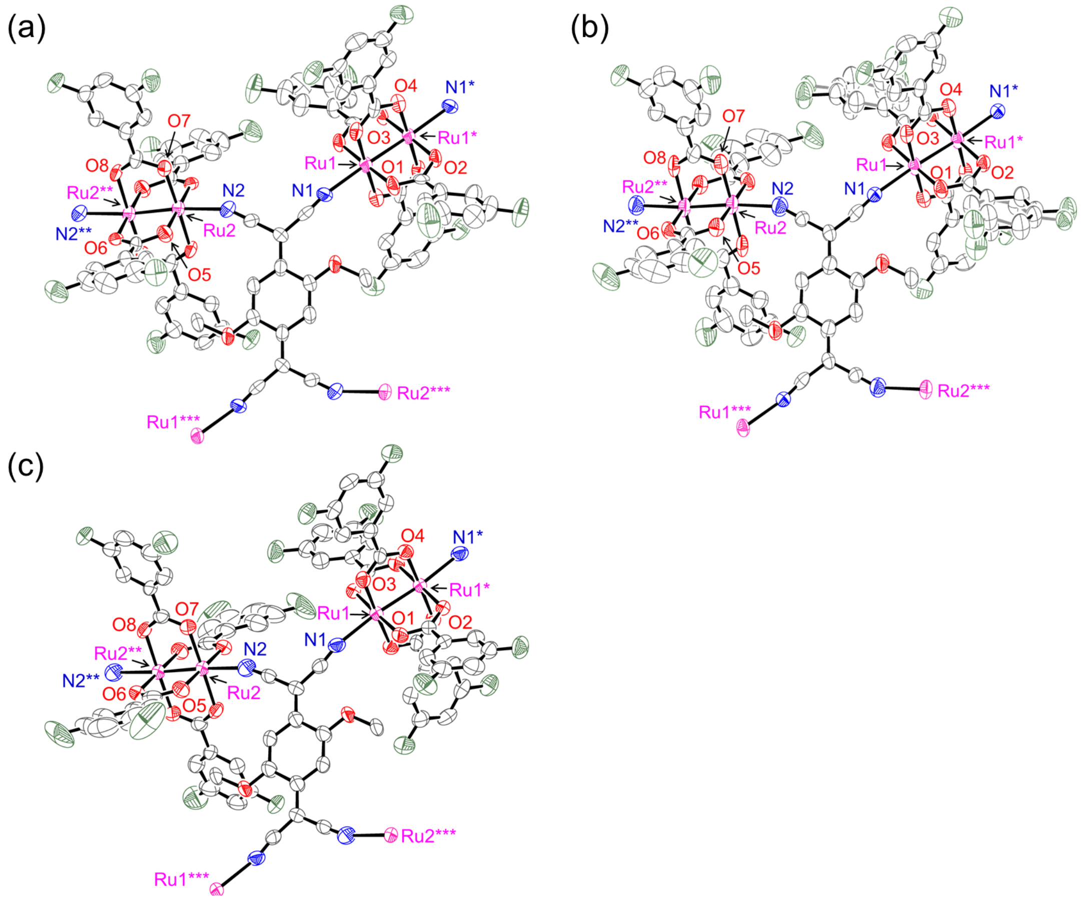

Supplementary Figure 6. Thermal ellipsoid plot obtained from SCXRD analyses showing the asymmetric unit and atom numbering scheme for **$1\supset N_2$** (a), **$1\supset O_2$-I** (b), and **$1\supset CO_2$** (c), where O, C, N, F, and Ru are represented in red, gray, blue, green, and purple, respectively. Displacement ellipsoids are drawn at a 50% probability level. Gray bond in (b) indicates the minor component of the rotational disorder. Hydrogen atoms and guest molecules are omitted for clarity. Symmetry codes: (*) $-x$, $-y$, $-z$, (**) $-x + 1$, $-y + 1$, $-z + 1$, (***) $-x$, $-y$, $-z + 1$.

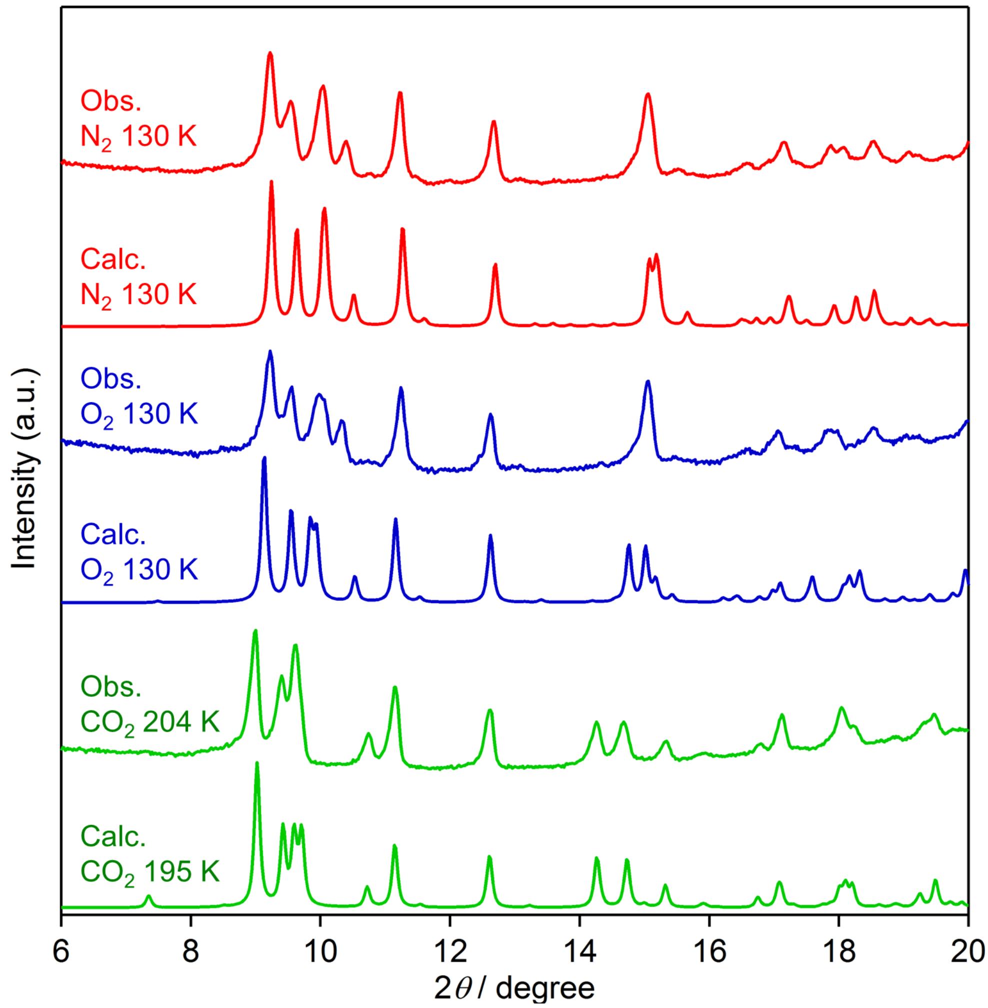

Supplementary Figure 7. Comparison of observed PXRD patterns and calculated PXRD patterns from the structures obtained from SCXRD analyses.

Supplementary Table 4. Crystallographic data obtained from SCXRD analyses for **1⊃N$_2$**, **1⊃O$_2$-I**, and **1⊃CO$_2$**.

	**1⊃N$_2$**	**1⊃O$_2$-I**	**1⊃CO$_2$**
formula	$C_{70}H_{32}F_{16}N_{12}O_{18}Ru_4$	$C_{70}H_{32}F_{16}N_4O_{28.3}Ru_4$	$C_{75}H_{32}F_{16}N_4O_{28}Ru_4$
formula weight	2037.35	2090.09	2145.34
crystal system	Triclinic	Triclinic	Triclinic
space group	$P-1$	$P-1$	$P-1$
a / Å	10.5642(18)	10.6878(15)	10.8173(11)
b / Å	11.986(2)	12.1373(17)	12.4400(12)
c / Å	15.718(3)	15.895(2)	15.9803(15)
α / deg	91.883(15)	91.728(12)	90.830(8)
β / deg	92.336(14)	93.681(12)	96.483(8)
γ / deg	103.024(16)	103.496(12)	104.721(9)
V / Å^3	1935.6(7)	1998.7(5)	2064.4(4)
Z	1	1	1
crystal size / mm^3	0.13×0.05×0.03	0.11×0.06×0.04	0.14×0.04×0.02
T / K	130(1)	130(1)	195(1)
D_{calc} / g·cm^{-3}	1.748	1.736	1.725
F_{000}	1000.00	1026.40	1054.00
λ / Å	0.71073	0.71073	0.71073
μ(Mo Kα) / cm^{-1}	8.799	8.610	8.359
data measured	12560	13334	13705
data unique	6894	7157	7394
R_{int}	0.0555	0.0512	0.0399
no. of observations	6894	7157	7394
no. of variables	541	632	615
$R1$ ($I > 2.00\sigma(I)$)^a	0.1194	0.1080	0.0808
R (all reflections)^a	0.1776	0.1814	0.1334
$wR2$ (all reflections)^b	0.3067	0.2893	0.2308
GOF	1.137	1.057	1.119
CCDC No.	1519243	1519244	1519240

^{a} $R1 = R = \Sigma||F_o| - |F_c||/\Sigma|F_o|$. ^{b} $wR2 = [\Sigma w(F_o^2 - F_c^2)^2/\Sigma w(F_o^2)^2]^{1/2}$

Supplementary Table 5. Selected bond length (Å) and angles (°) obtained from SCXRD analyses in **1⊃N$_2$**, **1⊃O$_2$-I**, and **1⊃CO$_2$**., where θ represents dihedral angle between the least squares planes defined by the phenyl ring of benzoate ligand and a carboxylate-bridging mode (atom set of M_2O_2C).

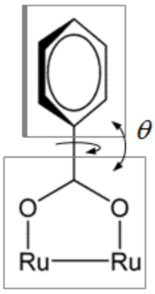

	**1⊃N$_2$**	**1⊃O$_2$-I**	**1⊃CO$_2$**.
Ru1–O1	2.001(11)	2.014(9)	2.036(7)
Ru1–O2a	2.019(10)	2.042(9)	2.049(7)
Ru1–O3	2.027(11)	2.023(13)	2.049(8)
Ru1–O4a	2.023(12)	2.038(11)	2.043(8)
Ru2–O5	2.032(12)	2.044(10)	2.027(8)
Ru2–O6b	2.041(12)	2.070(10)	2.029(8)
Ru2–O7	2.029(11)	2.021(12)	2.019(8)
Ru2–O8b	2.034(10)	2.053(11)	2.040(8)
Ru1–N1	2.207(14)	2.205(12)	2.229(10)
Ru2–N2	2.240(14)	2.246(13)	2.242(10)
Ru1–Ru1a	2.2710(18)	2.2911(16)	2.2879(11)
Ru2–Ru2b	2.2683(19)	2.2803(16)	2.2720(13)
Ru1–N1–C29	165.5(14)	164.5(12)	162.2(10)
Ru2–N2–C31	155.1(14)	154.5(13)	160.4(11)
θ			
benzoate set-1	29.52	20.57	13.93
benzoate set-2	2.91	2.32	6.25
benzoate set-3	10.78	9.43	10.55
benzoate set-4	11.21	14.48	16.34

Symmetry codes: (a) $-x, -y, -z$, (b) $-x + 1, -y + 1, -z + 1$.
Benzoate set-1 to -4: the phenyl group of C2–C8, C10–C16, C18–C24 and C26–C32, respectively.

Supplementary Note 5: On the oxidation state of the [Ru$_2$] unit in 1⊃N$_2$, 1⊃O$_2$-I, and 1⊃CO$_2$.

The oxidation state of [Ru$_2$] unit can be known from the Ru–O$_{eq}$ length (O$_{eq}$ = equatorial oxygen atoms), which is quite sensitive to the oxidation state of the [Ru$_2$] unit and to be 2.06–2.07 Å for [$Ru_2^{II,II}$] and 2.02–2.03 Å for [$Ru_2^{II,III}$]$^+$.[6] The average Ru–O$_{eq}$ length of **1⊃N$_2$** is 2.018 and 2.034 Å for [Ru(1)$_2$] and [Ru(2)$_2$] unit, respectively. The average Ru–O$_{eq}$ length of **1⊃O$_2$-I** is 2.029 and 2.047 Å for [Ru(1)$_2$] and [Ru(2)$_2$] unit, respectively. The average Ru–O$_{eq}$ length of **1⊃CO$_2$** is 2.044 and 2.029 Å for [Ru(1)$_2$] and [Ru(2)$_2$] unit, respectively.

Supplementary Table 6. Comparison of bond lengths (Å) obtained from SCXRD analyses in TCNQ moieties for **1⊃N₂**, **1⊃O₂-I**, and **1⊃CO₂**.

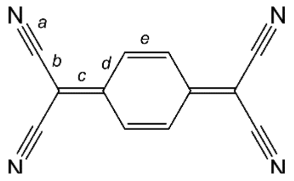

	charge	*a*	*b*	*c*	*d*	*e*	ρ^b	Ref
**I**	0	1.140(1)	1.441(1)	1.374(3)	1.448(4)	1.346(3)	0	7
**II**	−1	1.153(7)	1.416(8)	1.420(1)	1.423(3)	1.373(1)	−1	8
**1⊃N₂**		1.12(2)	1.41(2)	1.38(2)	1.46(2)	1.34(2)	−0.31[c]	
		1.14(2)	1.45(2)		1.39(2)			
		1.13[a]	1.43[a]		1.42[a]			
**1⊃O₂-I**		1.14(2)	1.45(2)	1.41(2)	1.45(2)	1.36(2)	−0.75[c]	
		1.160(18)	1.400(19)		1.41(2)			
		1.15[a]	1.42[a]		1.43[a]			
**1⊃CO₂**		1.158(16)	1.407(16)	1.402(17)	1.464(16)	1.356(18)	−0.71[c]	
		1.141(15)	1.417(16)		1.399(16)			
		1.150[a]	1.412[a]		1.432[a]			

I: TCNQ, **II**: RbTCNQ. [a]average value, [b]estimated from the average values, [c]$\rho = A_H[c/(b + d)] + B_H$ with $A_H = -41.667$ and $B_H = 19.833$.

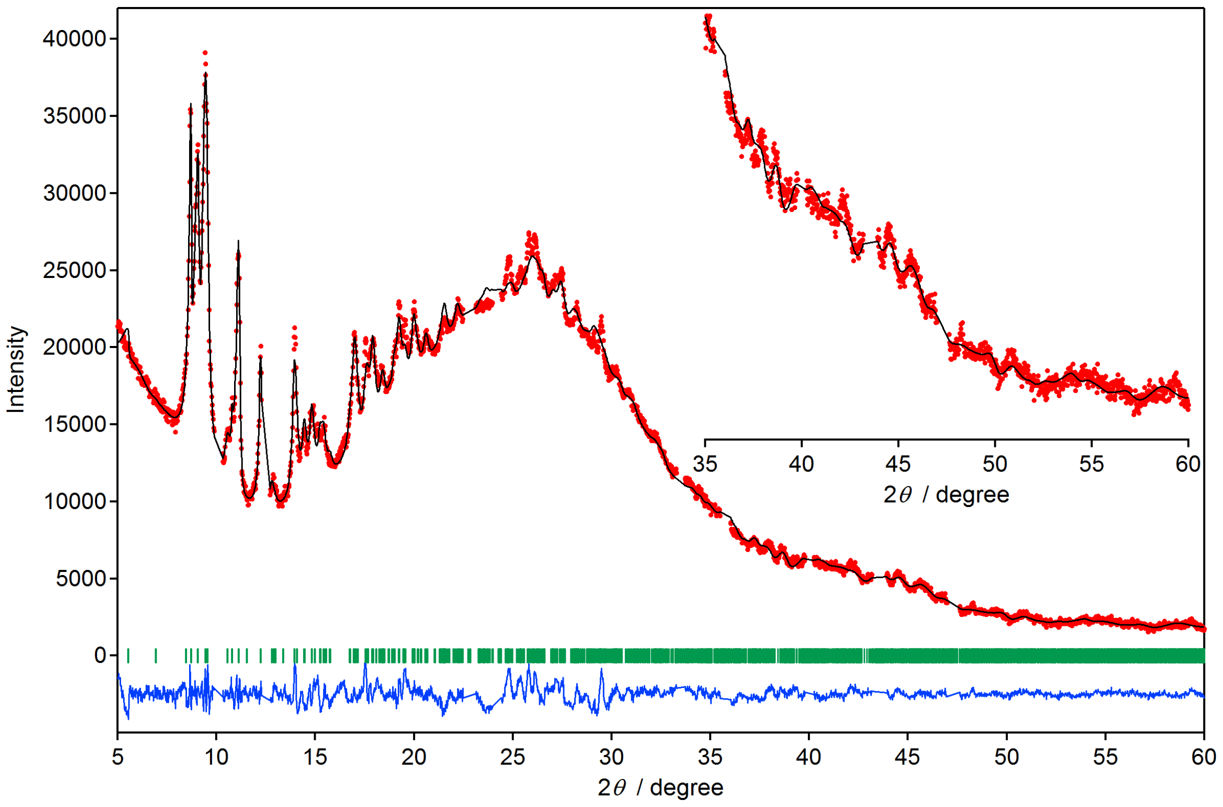

Supplementary Figure 8. PXRD pattern (λ = 1.54 Å) and Rietveld analysis for **1⊃O₂** at 94 K and P_{O2} = 100 kPa. Red dots, black line, and blue line are the observed plots, calculated pattern, and their difference, respectively. Green bars represent the calculated positions of the Bragg reflections.

Supplementary Table 7 Crystallographic data obtained from Rietveld refinement for **1⊃O₂-II**

	**1⊃O_2-II**
formula	$C_{70}H_{32}F_{16}N_4O_{34}Ru_4$
formula weight	2181.264
crystal system	Triclinic
space group	P−1
a / Å	10.697(2)
b / Å	12.910(2)
c / Å	15.826(2)
α / deg	90.225(14)
β / deg	98.05(2)
γ / deg	104.031(16)
V / Å^3	2097.8(7)
Z	1
T / K	94(1)
D_{calc} / g·cm^{-3}	1.727
F_{000}	1072.00
λ / Å	1.5406
$2\theta_{max}$ / deg	60.00
$2\theta_{min}$ / deg	5.00
step size / deg	0.02
R_{wp} [a]	0.03313
R_B [b]	0.01219
CCDC No.	1519245

[a] $R_{wp} = [\sum w[y - f(\boldsymbol{x})]^2/\sum wy^2]^{1/2}$, where y and $f(\boldsymbol{x})$ represent the observed intensity and the calculated intensity at a diffraction angle of 2θ, respectively. [b] $R_B = \sum||F_o| - |F_c||/\sum|F_o|$.

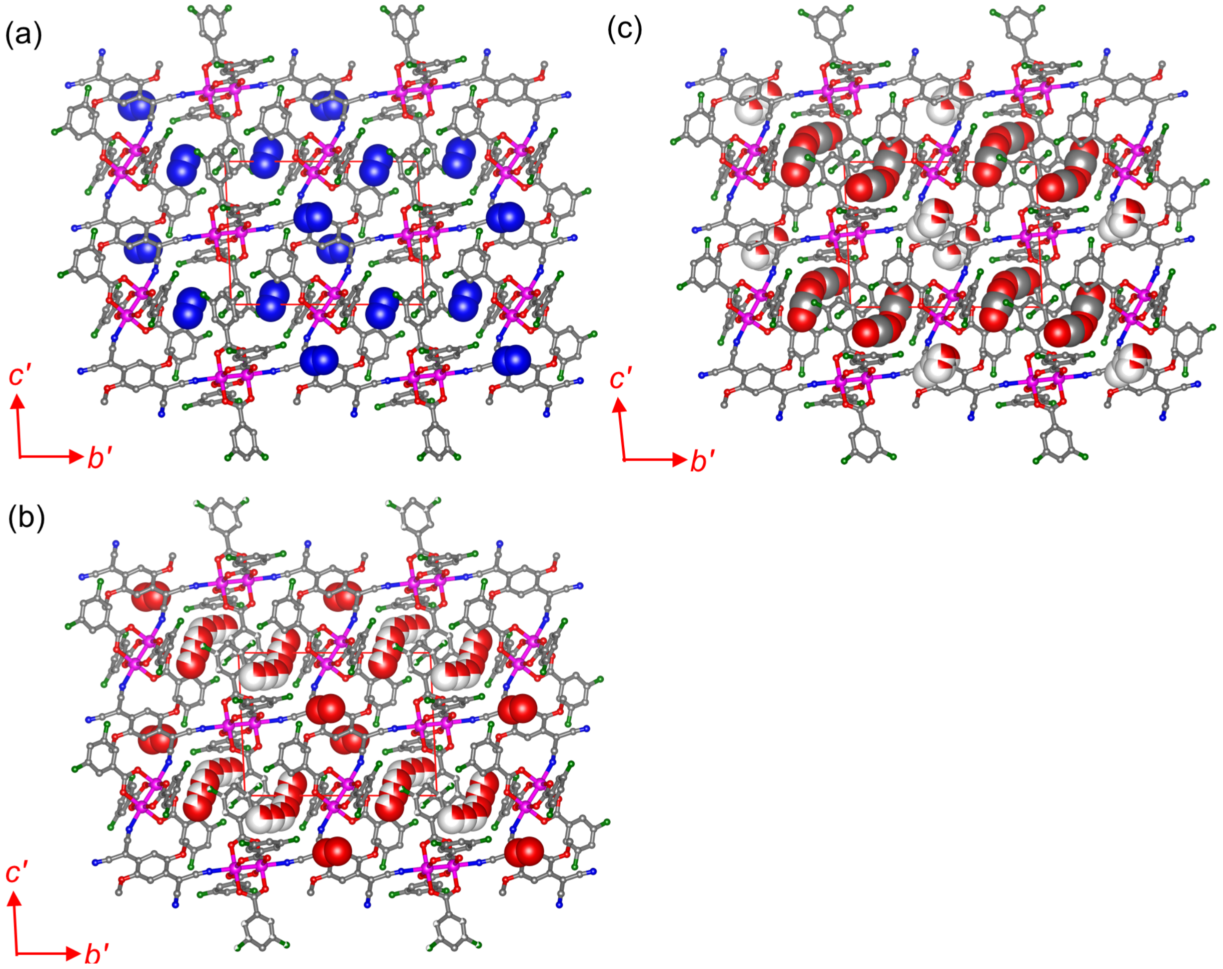

Supplementary Figure 9. Packing views of **1⊃N_2** (a), **1⊃O_2-I** (b), and **1⊃CO_2** (c) obtained from SCXRD analyses along the *a*′-axis, where atoms N, O, C, F and Ru are represented in blue red, gray, green, and purple, respectively. The adsorbed gas molecules that were located by the structural analyses are given in space filling model, where the colored area fraction of the atomic sphere corresponds to site occupancy. The axes in red represent a transformed lattice for an easy comparison with the lattice of **1-solv** (see Supplementary Methods). Minor component of positional disordered atoms of the benzoate moiety in **1⊃O_2-I** were omitted for clarity.

Supplementary Note 6: Additional description on the crystal structure of 1⊃Gas.

The crystal structures of **1⊃Gas** phases were determined based on SCXRD (**1⊃N_2**, **1⊃O_2-I**, and **1⊃CO_2**) or PXRD data using the Rietveld refinement technique (**1⊃O_2-II**). For the construction of the plausible initial structural model of **1⊃O_2-II** that satisfies the observed PXRD pattern by a direct-space method, the introduction of gas molecules into the frameworks is absolutely imperative, although the assigned numbers and positions of gas molecules may not be entirely accurate. And thus, only the framework structure is discussed for **1⊃O_2-II** although adsorbed O_2 molecules are reported in CIF file. The fishnet-like layered framework is preserved for all **1⊃Gas** phases, which lie on the (1−10) plane but upon gas adsorption the environment between layers of **1⊃Gas** largely varied. For **1⊃N_2**, **1⊃O_2-I**, and **1⊃CO_2**, some of the adsorbed gases could be located at the fixed positions, and some could not due to disorder. The correspondence between the solved structures and observed PXRD patterns are shown in Supplementary Fig. 7. The slight difference between calculated and observed pattern under O_2 at 130 K indicate a little difference in the lattice constant for powder sample and single crystal.

Upon the structural change, π-stacks between the phenyl rings on the benzoate of both (**1⊃CO_2**) or one of the two (**1⊃N_2** and **1⊃O_2-I**) [Ru_2] units also recovered (distances between π-planes on [$Ru(1)_2$] and [$Ru(2)_2$] units in **1⊃CO_2** were 3.16 and 3.22 Å, respectively, and distances between π-planes on [$Ru(2)_2$] units in **1⊃N_2** and **1⊃O_2-I** were 3.20 and 3.25 Å, respectively), while there is no inter-layer interaction through π-stack in **1⊃O_2-II**.

Supplementary Note 7: Additional description on the gas-adsorption site for 1⊃N_2, 1⊃O_2-I, and 1⊃CO_2 determined by SCXRD structural analyses

Gas-accommodation site can be regarded as common among the three phases, and classified into three sites (Supplementary Fig. 10); site-A and site-B are adjacent to π-plane and methoxy group of TCNQ(MeO)$_2$, respectively, while site-C is located between site-B. In other words, site-A and site-C are the pores formed between [Ru_2]-TCNQ fishnet layers, while site-B locates at the center of the fishnet.

Judging from site occupancy in SCXRD structural analyses, N_2 and O_2 gases prefer site-A, while disordered CO_2 with small site occupancy was found at site-A. Conversely, CO_2 gases prefer site-B and -C. N_2 gases are also found at site-B, though, not at site-C. Disordered O_2 gases are distributed among site-B and -C.

Relevant structural parameters around site-A were shown in Fig. 2d for **1⊃O_2-I** and **1⊃N_2** phases. Judging from the location of gases, paramagnetic O_2 gases at site-A are expected to be involved in magnetic interaction pathway, while diamagnetic N_2 gases cannot. To discuss the magnetic interaction through O_2 gases, further investigations are required, such as temperature, pressure dependence of SCXRD study and theoretical calculation.

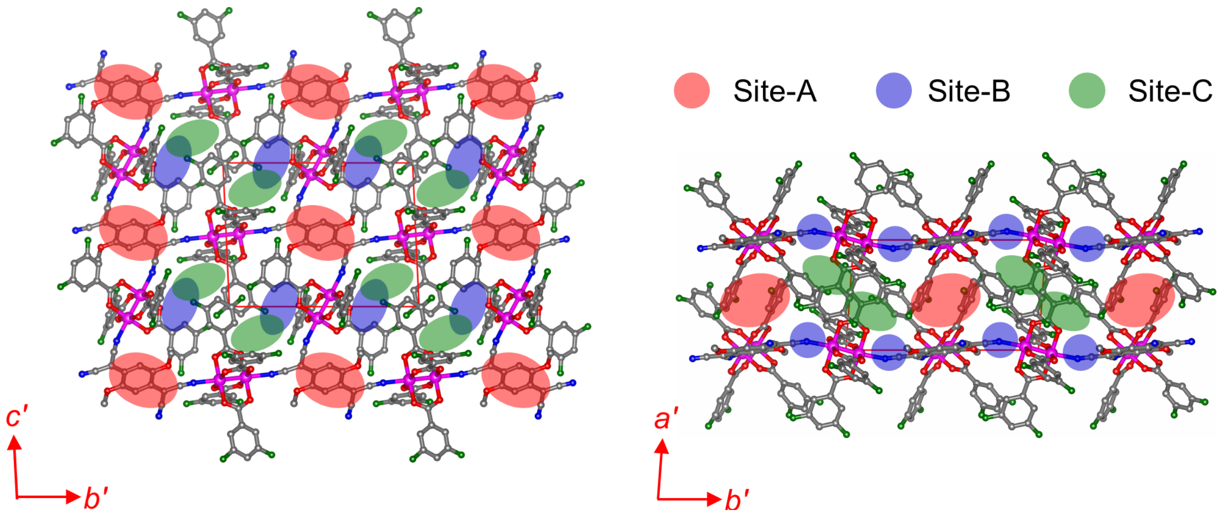

Supplementary Figure 10. Schematic diagram of gas adsorption sites (Site-A–C) for **1⊃N_2**, **1⊃O_2-I**, and **1⊃CO_2** along the *a*′-axis (left) and the *c*′-axis (right). The axes in red represent a transformed lattice for an easy comparison with the lattice of **1-solv** (see Supplementary Methods).

Supplementary Table 8. Relevant structural data for **1-solv**, **1**, **1⊃N_2**, **1⊃O_2-I**, **1⊃O_2-II**, and **1⊃CO_2**

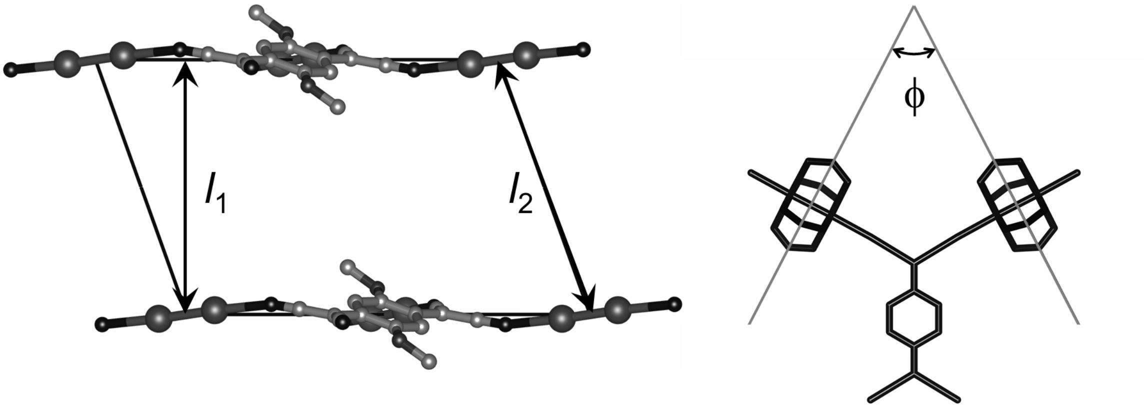

	**1-solv**	**1**	**1⊃N_2** (130 K)	**1⊃O_2-I** (130 K)	**1⊃O_2-II** (94 K)	**1⊃CO_2** (204 K)
Vertical distance between layers l_1 (Å)	9.78	9.46	8.76	8.89	9.11	9.10
[Ru_2]–[Ru_2] Distance between layers (inter-unit translational distance) l_2 (= a'/Å)	10.65	10.84	10.56	10.69	10.70	10.82
Guest molecules accessible volume or void volume (Å^3)	713.1 (32.0 %)	147.3 (7.5 %)	335.3 (17.3 %)	369.3 (18.5 %)	546.1 (26.0 %)	447.9 (21.7 %)
Ru1–N1–C29 (°)	154.7	132.2	165.5	164.5	153.1	162.2
Ru2–N2–C31 (°)	165.4	148.3	155.1	154.5	165.5	160.4
Av. Ru–N–C (°)	160.05	140.25	160.3	159.5	159.3	161.3
Angle between paddlewheels (ϕ/°)	35.9	49.4	47.6	49.3	45.8	52.9
Number of adsorbed molecule (observed)	3DCM·1.5DCE	–	2.3N_2 (120 K)	5.5O_2 (120 K)	9.5O_2 (90 K)	8.7CO_2 (195 K)
Number of adsorbed molecule (located by crystallographc analysis)	3DCM·1.5DCE	–	4N_2	5.2O_2	8O_2	5CO_2
Analytical method	SCXRD	SCXRD	SCXRD	SCXRD	Rietveld[a]	SCXRD

[a] The Rietveld technique based on a PXRD pattern

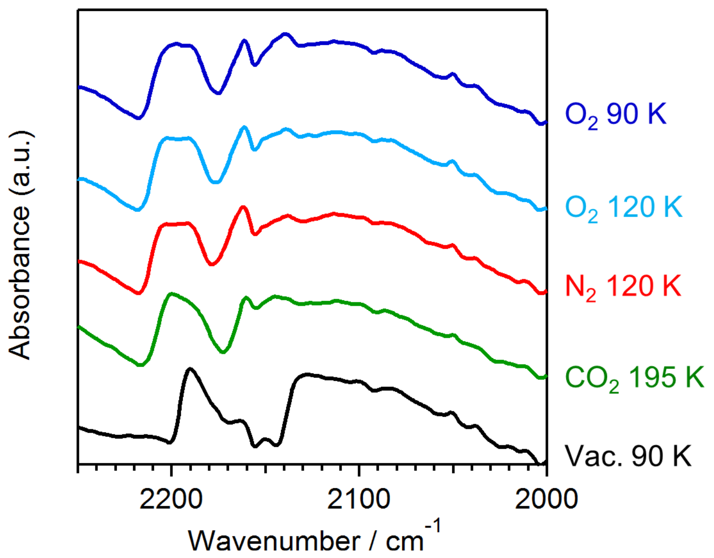

Supplementary Figure 11. IR spectra of **1** measured under 100 kPa of O_2 at 90K (blue) and at 120 K (cyan), N_2 at 120 K (red), CO_2 at 195 K and vacuum at 90 K (black).

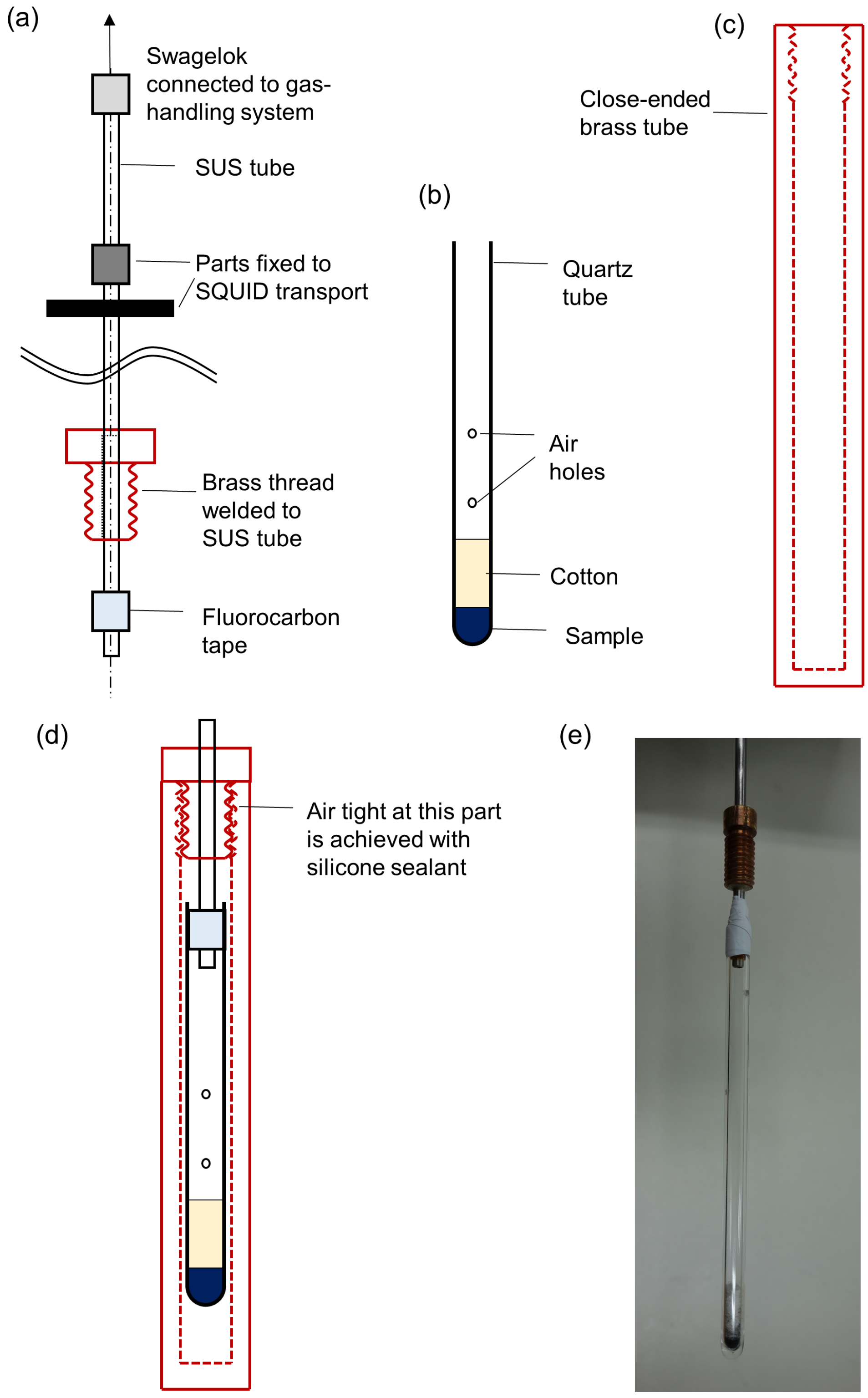

Supplementary Figure 12. Scheme of the cell for *in situ* magnetization measurement under gas; sample rod (a), quartz tube (b), outer brass tube (c) and assembling drawing (d). Picture of *in situ* cell without outer brass tube.

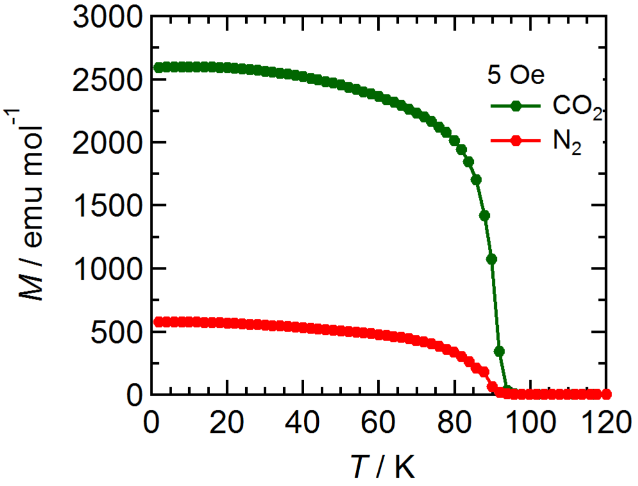

Supplementary Figure 13. FCM curves at 5 Oe for **1⊃CO₂** (green) and **1⊃N₂** (red).

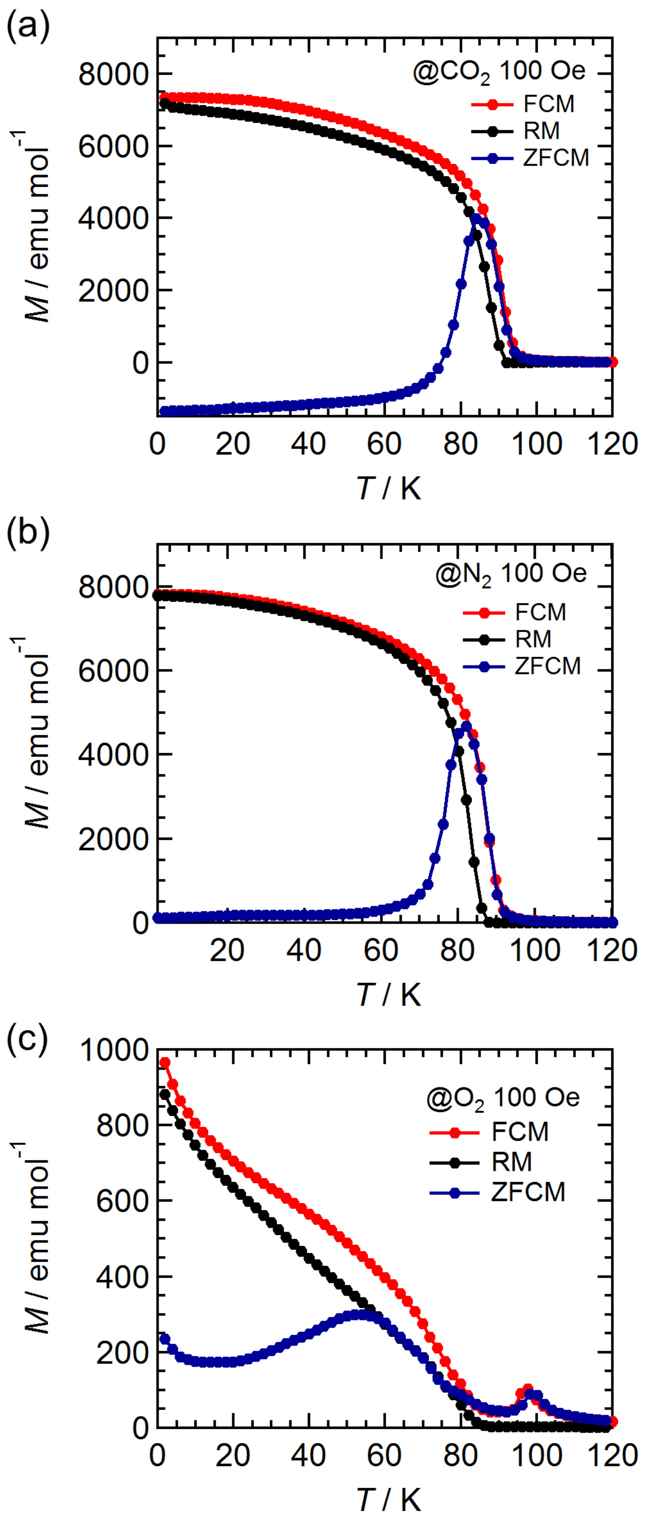

Supplementary Figure 14. FCM (red), RM (green), and zero-field-cooled magnetization (ZFCM, blue) curves at 100 Oe for **1⊃CO_2** (a), **1⊃N_2** (b), and **1⊃O_2** (c).

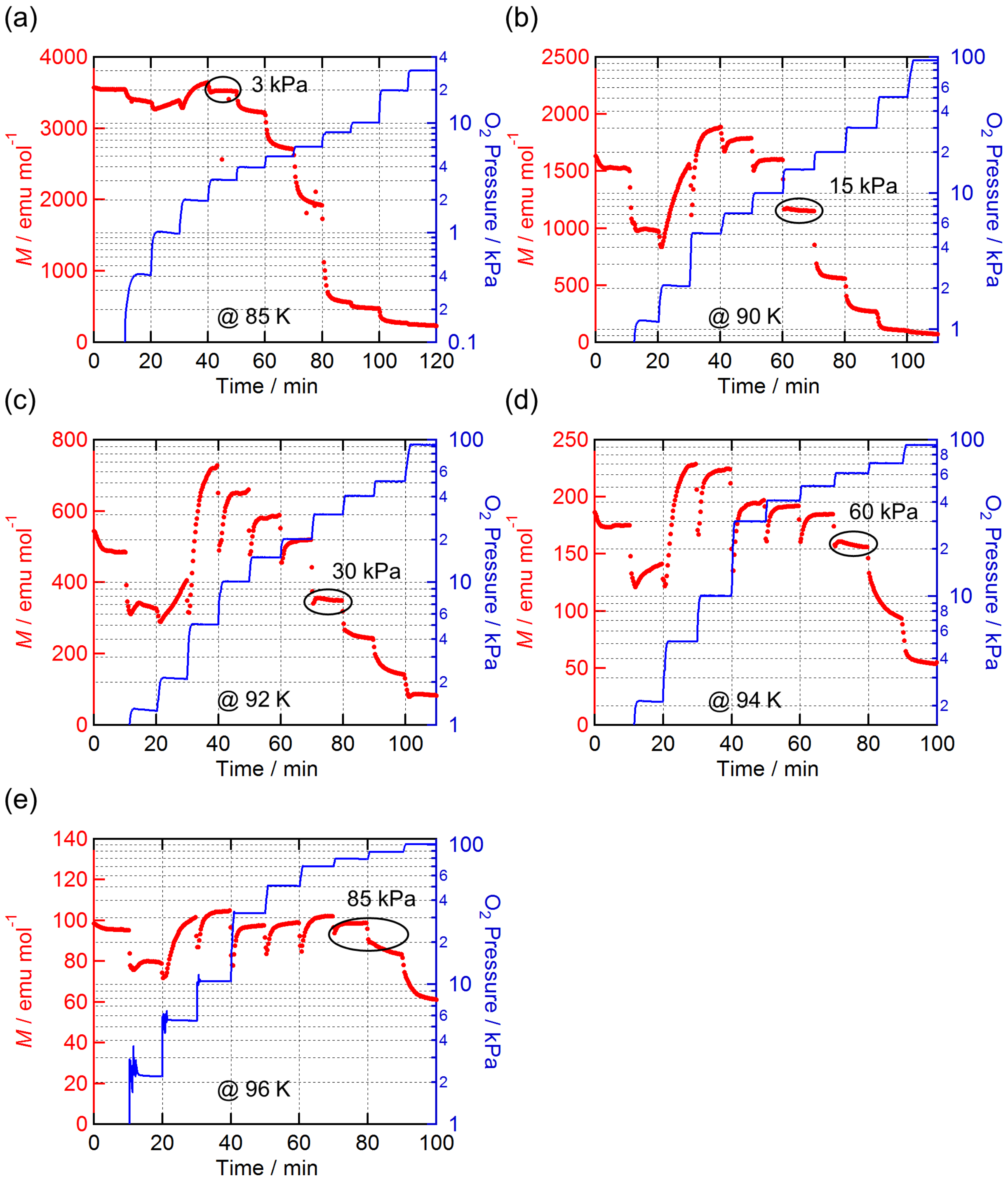

Supplementary Figure 15. The magnetization measurements via O_2-pressure variation at each temperature; 85 K (a), 90 K (b), 92 K (c), 94 K (d), and 96 K (e). Because the introduction of O_2 into sample cell causes slight increase in sample temperature, the following magnetic response sensitively indicates that the present temperature (T) is above or below Neel temperature (T_N); when increase, $T > T_N$ while when decrease, $T < T_N$. When $T = T_N$, the response should be almost constant, which was indicated by black circle in the figures with the value of applied O_2-pressure.

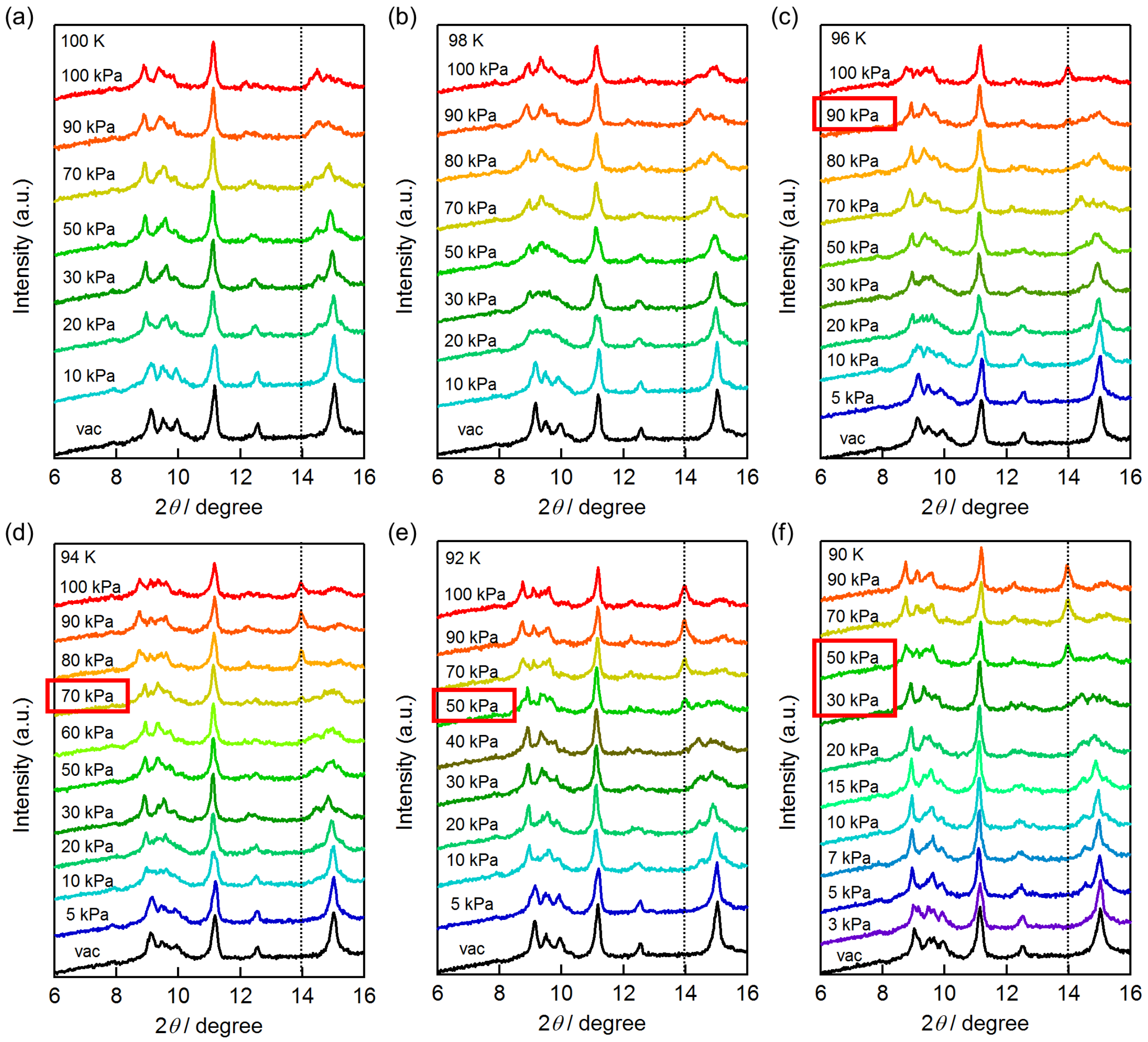

Supplementary Figure 16. The PXRD patterns (θ–2θ scan, λ = 1.54 Å) measured by varying O_2-pressure at each temperature; 100 K (a), 98 K (b), 96 K (c), 94 K (d), 92 K (e), and 90 K (f). The appearance of **1⊃O_2-II** was monitored by the presence of peak at 13.96°, which was indicated by dotted line in the figures. The transition O_2-pressures from **1⊃O_2-I** to **1⊃O_2-II** at each temperature are indicated by red squares in the figures.

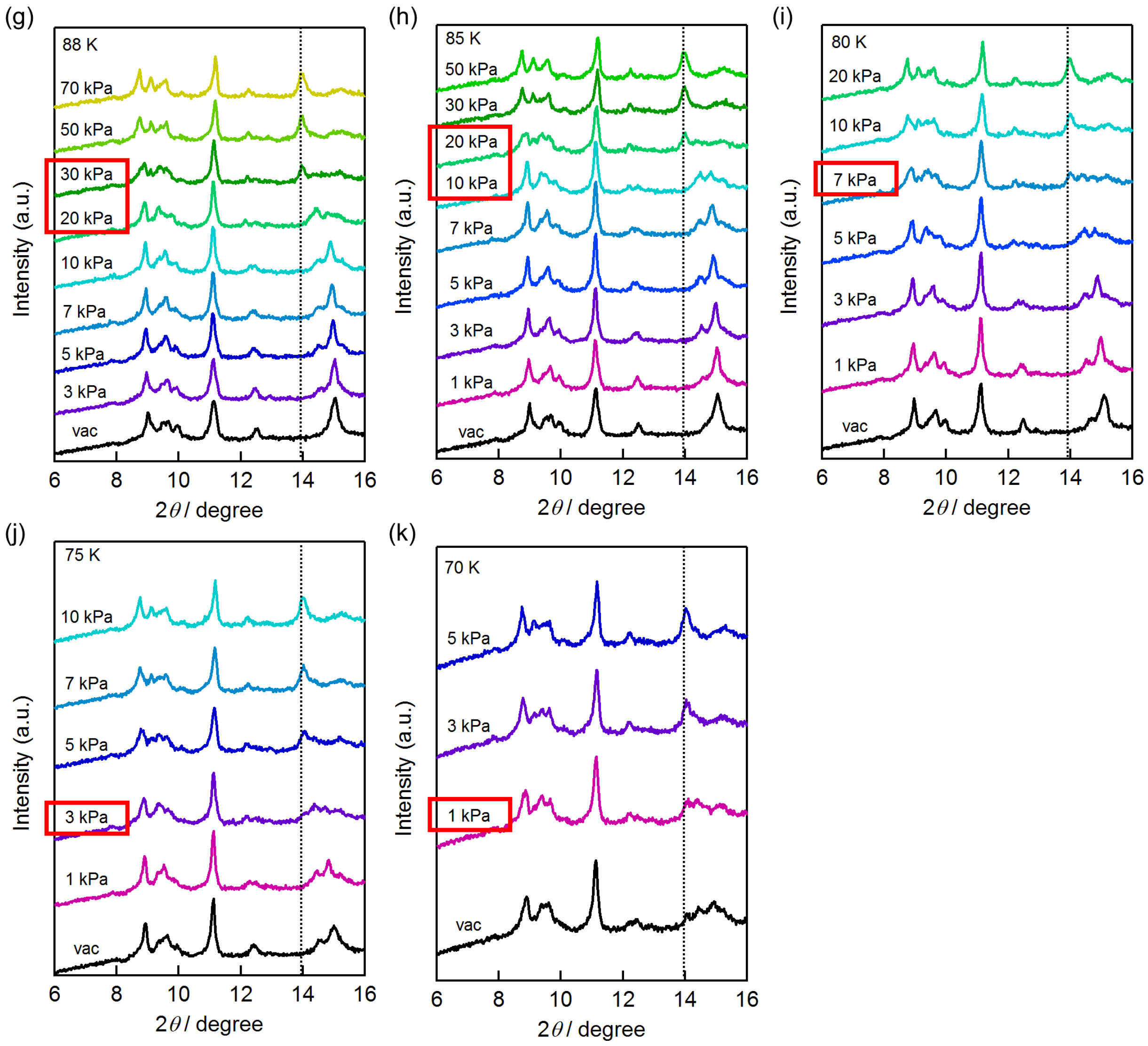

Supplementary Figure 16 (continued). The PXRD patterns (θ–2θ scan, λ = 1.54 Å) measured by varying O_2-pressure at each temperature; 88 K (g), 85 K (h), 80 K (i), 75 K (j), and 70 K (k). The appearance of **1⊃O_2-II** was monitored by the presence of peak at 13.96°, which was indicated by dotted line in the figures. The transition O_2-pressures from **1⊃O_2-I** to **1⊃O_2-II** at each temperature are indicated by red squares in the figures.

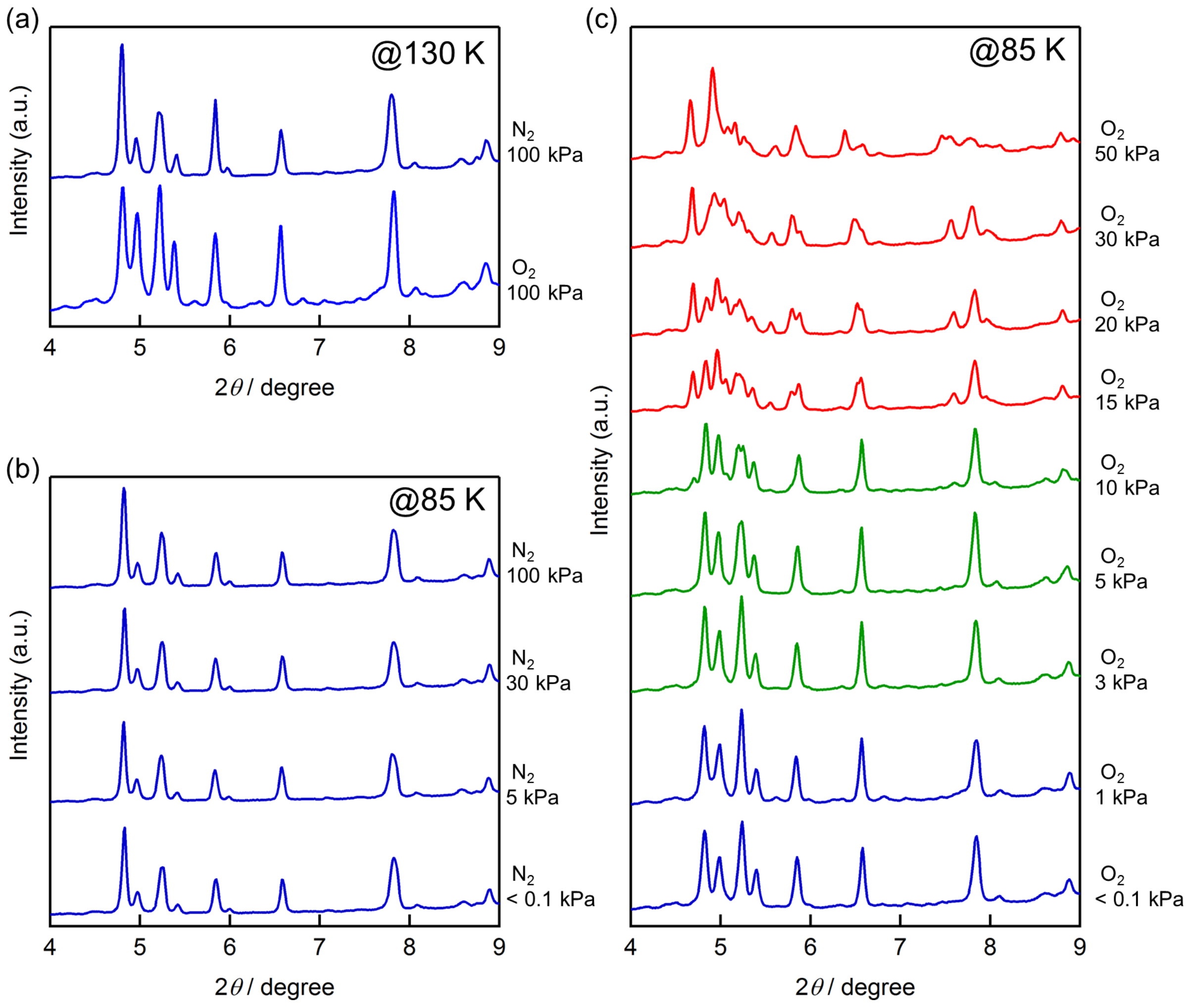

Supplementary Figure 17. The high resolution synchrotron PXRD patterns (λ = 0.80 Å). (a) Measured at 130 K under 100 kPa of N_2 and O_2, where **1⊃N₂** and **1⊃O₂-I** phases were observed, respectively, which are isostructural. (b) Measured by varying N_2-pressure at 85 K, indicating that the structures remain unchanged. (c) Measured by varying O_2-pressure at 85 K. Blue, green, red lines indicate ferrimagnet in **1⊃O₂-I** phase, antiferromagnet in **1⊃O₂-I** phase, and antiferromagnet in **1⊃O₂-II** phase, respectively.

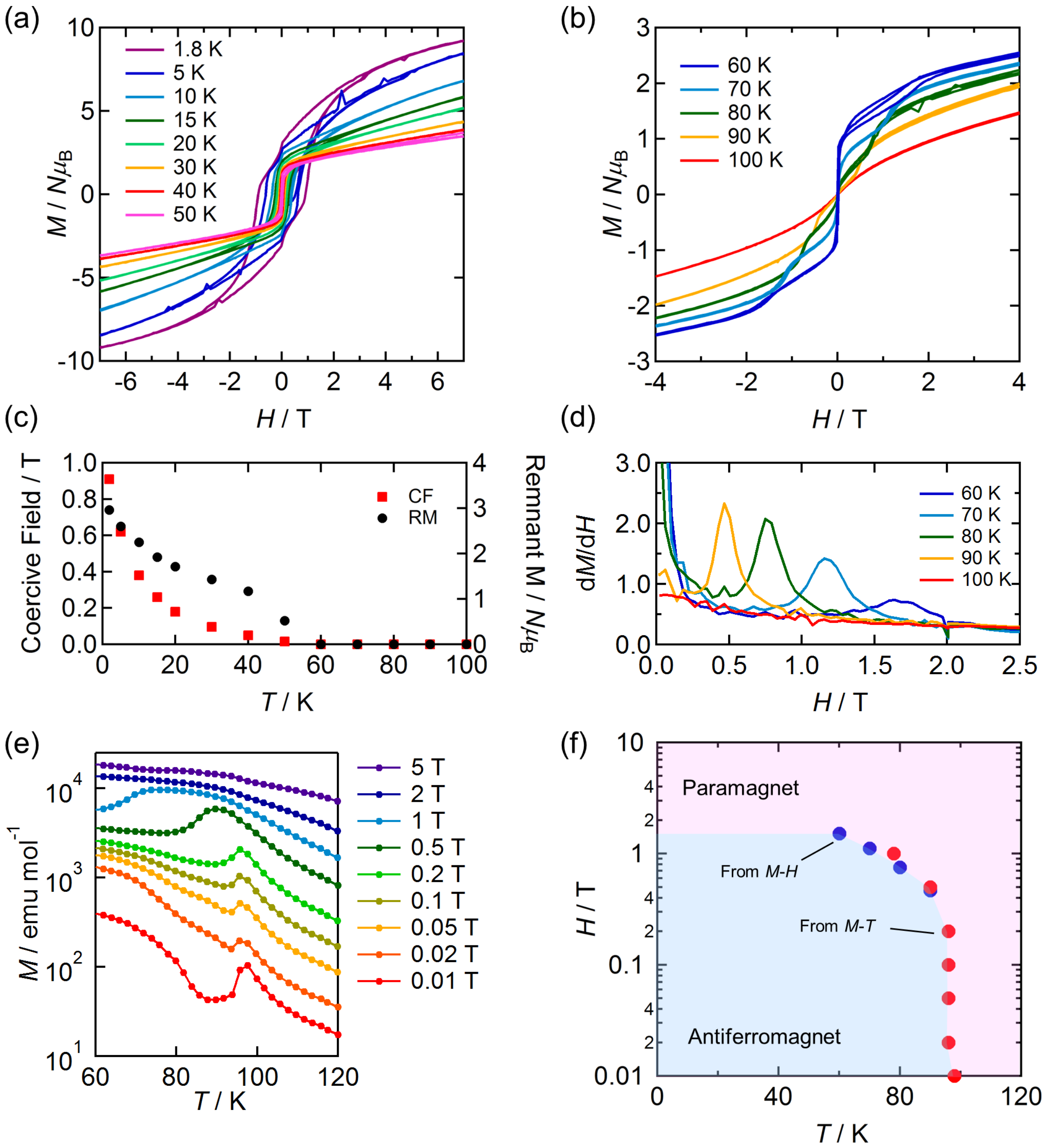

Supplementary Figure 18. Magnetic properties for **1⊃O₂** (P_{O2} = 100 kPa). Temperature dependence of *M*–*H* curves for 1.8−50 K (a) and 60−100 K (b), temperature dependence of coercive field and remnant magnetization (c), d*M*/d*H* vs *H* plots for the virgin magnetization (d), FCM curves measured under different external fields (e), and magnetic phase diagram determined from *M*–*T* curves (red dot) and *M*–*H* curves (blue dot).

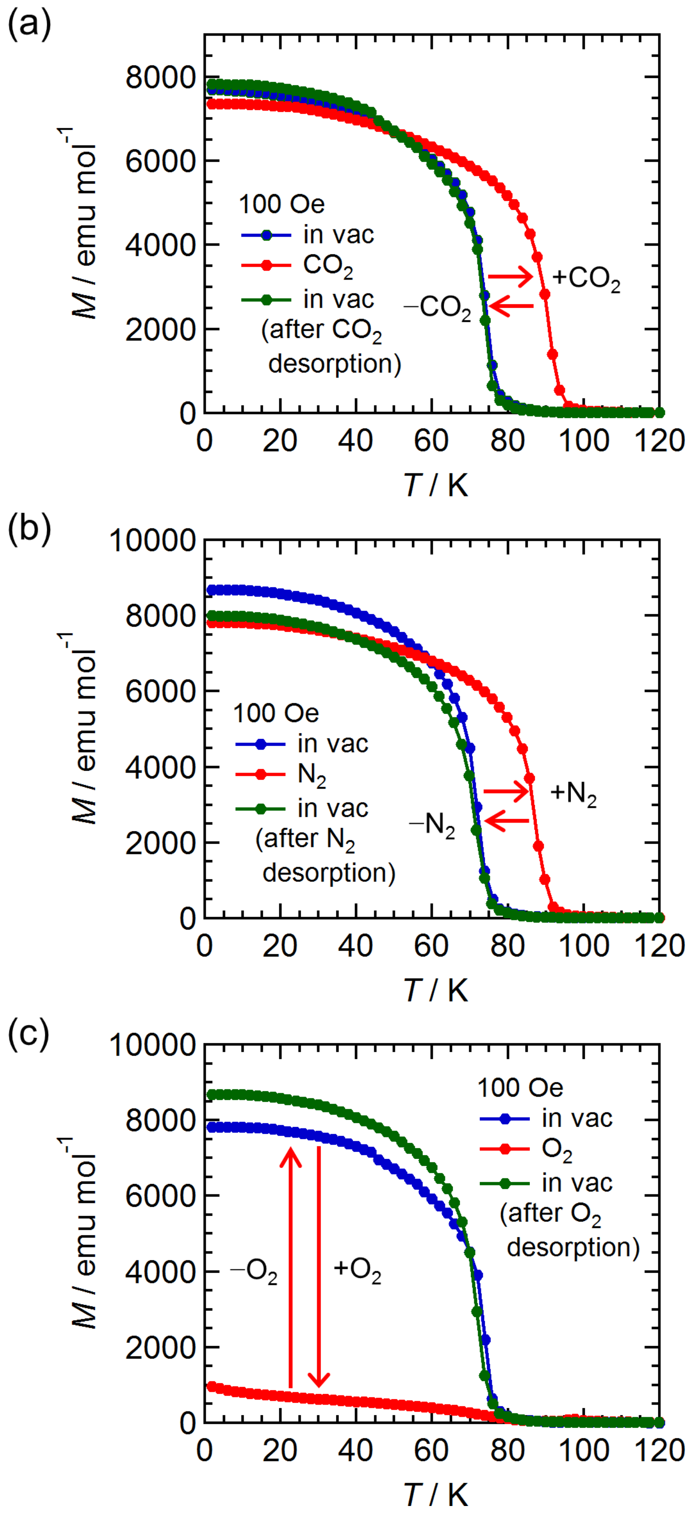

Supplementary Figure 19. FCM curves at 100 Oe for **1** before gas introduction (blue), **1⊃Gas** (red) and **1⊃Gas** after gas desorption (green); CO_2 (a), N_2 (b) and O_2 (c).

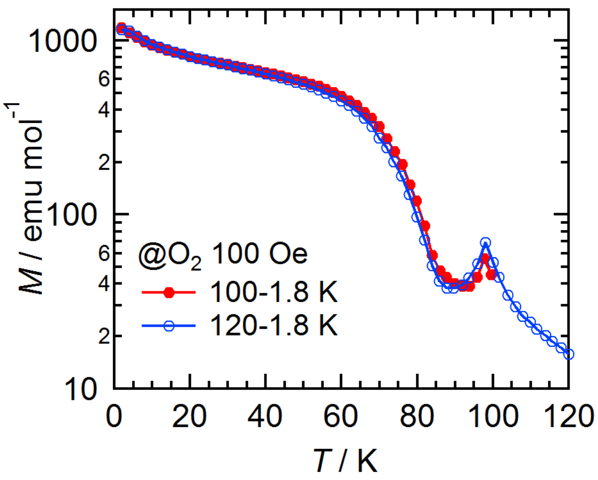

Supplementary Figure 20. FCM curves at 100 Oe for **1⊃O₂** between 100−1.8 K (red) and 120−1.8 K (blue).

Supplementary References

1 Miyasaka, H., Motokawa, N., Atsuumi, R., Kamo, H., Asai, Y. & Yamashita, M. Tuning of the ionization potential of Paddlewheel diruthenium(II,II) complexes with fluorine atoms on the benzoate ligands. *Dalton Trans.* **40**, 673−682 (2011).

2 Momma, K. & Izumi, F. VESTA 3 for three-dimensional visualization of crystal, volumetric and morphology data. *J. Appl. Cryst.* **44**, 1272−1276 (2011).

3 van der Sluis, P. & Spek, A. L. BYPASS: an effective method for the refinement of crystal structures containing disordered solvent regions. *Acta Cryst. Sec. A* **46**, 194−201 (1990).

4 Favre-Nicolin, V. & Černý, R. *FOX*, 'free objects for crystallography': a modular approach to *ab initio* structure determination from powder diffraction. *J. Appl. Cryst.* **35**, 734−743 (2002).

5 Izumi, F &.Momma, K. Three-dimensional Visualization in Powder Diffraction. *Solid State Phenom.* **130**, 15−20 (2007).

6 Cotton, F. A.& Walton, R. A. *Multiple Bonds Between Metal Atoms, 2nd ed.*, (Oxford University Press, Oxford, 1993).

7 Long, R. E., Sparks, R. A. & Trueblood, K. N. The Crystal and Molecular Structure of 7,7,-8,8-Tetracyanoquinodimethane. *Acta Cryst.* **18**, 932−939 (1965).

8 Fritche, C. J., Jr. & Arthur, P., Jr. A Refinement on the Crystal Structure of Cesium Tetracyanoquinodimethanide. *Acta Cryst.* **21**, 139−145 (1966).
